# Predictive Value of Precision-Cut Kidney Slices as an Ex Vivo Screening Platform for Therapeutics in Human Renal Fibrosis

**DOI:** 10.3390/pharmaceutics12050459

**Published:** 2020-05-18

**Authors:** Emilia Bigaeva, Nataly Puerta Cavanzo, Elisabeth G. D. Stribos, Amos J. de Jong, Carin Biel, Henricus A. M. Mutsaers, Michael S. Jensen, Rikke Nørregaard, Anna M. Leliveld, Igle J. de Jong, Jan-Luuk Hillebrands, Harry van Goor, Miriam Boersema, Ruud A. Bank, Peter Olinga

**Affiliations:** 1 Department of Pharmaceutical Technology and Biopharmacy, University of Groningen, Groningen Research Institute of Pharmacy, 9713 AV Groningen, The Netherlands; e.bigaeva@rug.nl (E.B.); n.puerta.cavanzo@rug.nl (N.P.C.); isabel.stribos@gmail.com (E.G.D.S.); amosjongde@gmail.com (A.J.d.J.); c.biel@rug.nl (C.B.); h.a.m.mutsaers@rug.nl (H.A.M.M.); m.boersema@umcg.nl (M.B.); 2 Department of Pathology and Medical Biology, University of Groningen, University Medical Center Groningen, 9713 GZ Groningen, The Netherlands; j.l.hillebrands@umcg.nl (J.-L.H.); h.van.goor@umcg.nl (H.v.G.); r.a.bank@umcg.nl (R.A.B.); 3 Department of Internal Medicine, Division of Nephrology, University of Groningen, University Medical Center Groningen, 9713 GZ Groningen, The Netherlands; 4 Department of Clinical Medicine, Aarhus University, 8200 Aarhus N, Denmark; msj@clin.au.dk (M.S.J.); rn@clin.au.dk (R.N.); 5 Department of Urology, University of Groningen, University Medical Center Groningen, 9713 GZ Groningen, The Netherlands; a.m.leliveld@umcg.nl (A.M.L.); i.j.de.jong@umcg.nl (I.J.d.J.)

**Keywords:** renal fibrosis, precision-cut kidney slices, antifibrotic drugs, pirfenidone, galunisertib, imatinib

## Abstract

Animal models are a valuable tool in preclinical research. However, limited predictivity of human biological responses in the conventional models has stimulated the search for reliable preclinical tools that show translational robustness. Here, we used precision-cut kidney slices (PCKS) as a model of renal fibrosis and investigated its predictive capacity for screening the effects of anti-fibrotics. Murine and human PCKS were exposed to TGFβ or PDGF pathway inhibitors with established anti-fibrotic efficacy. For each treatment modality, we evaluated whether it affected: (1) culture-induced collagen type I gene expression and interstitial accumulation; (2) expression of markers of TGFβ and PDGF signaling; and (3) expression of inflammatory markers. We summarized the outcomes of published in vivo animal and human studies testing the three inhibitors in renal fibrosis, and drew a parallel to the PCKS data. We showed that the responses of murine PCKS to anti-fibrotics highly corresponded with the known in vivo responses observed in various animal models of renal fibrosis. Moreover, our results suggested that human PCKS can be used to predict drug efficacy in clinical trials. In conclusion, our study demonstrated that the PCKS model is a powerful predictive tool for ex vivo screening of putative drugs for renal fibrosis.

## 1. Introduction

Animal models are powerful tools in the study of disease mechanisms and preclinical development of therapeutics. However, modeling human disease, such as chronic kidney disease (CKD), is a very challenging task [1]. CKD is a major global health concern characterized by the progressive loss of renal function [2]. Irrespective of etiology, renal fibrosis is a driving force in the progression of CKD and it is often regarded as the most damaging process in kidney disease [3,4]. Currently, treatment options for CKD are limited to blood pressure regulation by renin angiotensin-aldosterone-system (RAAS) blockade, which only delay the decline in renal function [5]. Despite overwhelming research efforts, no pharmacological intervention is currently available that effectively halts the progression of renal fibrosis in CKD patients.

The molecular processes involved in human renal fibrosis are extremely complex, and the deposition of extracellular matrix (ECM) proteins is orchestrated by numerous cells, including (myo)fibroblasts, pericytes, fibrocytes, tubular epithelial cells, endothelial cells, inflammatory cells, and resident and infiltrating stem cells [6,7,8]. Historically, transforming growth factor beta (TGFβ) has been regarded as the master regulator of fibrosis, which promotes matrix synthesis and myofibroblast activation [9,10]. Other growth factors, particularly platelet-derived growth factor (PDGF), have also been implicated in renal fibrosis. PDGF is a potent inducer of proliferation, differentiation, and migration of fibrogenic mesenchymal cells [11,12]. Given their central role in renal fibrosis, the TGFβ and PDGF pathways are important therapeutic targets [13,14,15].

Despite many successful preclinical studies, only limited advances have been made in the translation of these findings to the level of patient treatment. Insufficient predictive capability of current animal models of renal fibrosis and lack of translational models for human disease contribute to the discrepancies between preclinical and clinical evidence [16]. The ex vivo model of precision-cut kidney slices (PCKS) has the potential to fill this gap. Several studies have successfully demonstrated the use of PCKS as a tool to study the development of fibrosis and screen the efficacy of anti-fibrotic treatments [17,18,19,20,21]. PCKS retain the native three-dimensional architecture of whole kidneys and preserve cell–cell signaling pathways that are lost in isolated cell culture, although the latter greatly facilitates basic research. A particular advantage of PCKS is that they can be prepared directly from human tissue [22], thereby removing cross-species heterogeneity in tissue responses to injury and drug intervention.

The aim of this study was to demonstrate the predictive value of PCKS as a screening platform for the anti-fibrotic activity of drug candidates. To this end, we prepared PCKS from healthy and fibrotic, murine, and human kidneys and evaluated the impact of three compounds with established anti-fibrotic efficacy—pirfenidone, galunisertib, and imatinib. Pirfenidone is a small synthetic molecule that was approved for the treatment of idiopathic pulmonary fibrosis. The compound exerts anti-fibrotic and anti-inflammatory activity in a variety of animal and cell-based models, and its effects seem to be associated with inhibition of the TGFβ pathway, although the specific mechanism remains largely unknown [23]. Galunisertib (LY2157299 monohydrate), a small molecule kinase inhibitor, selectively blocks TGFβ receptor I (ALK5), thereby inhibiting canonical SMAD2 signaling [24]. Galunisertib is mostly known as an anti-cancer agent, and is currently in phase II multi-site trials for the treatment of hepatocellular carcinoma (NCT01246986), yet there is growing preclinical evidence for its anti-fibrotic activity [25,26,27]. Imatinib (also known as STI571 or Gleevec) is a multi-kinase inhibitor of the c-abl, c-kit, and PDGFR tyrosine kinases [28], which is commonly used in the management of leukemia and mesenchymal tumors, and has been shown to diminish hepatic and renal fibrosis in vivo [29,30,31,32]. In addition, we investigated whether ex vivo PCKS responses to anti-fibrotics correlate with the responses of activated (myo)fibroblasts in vitro, considering that the latter are the principle effector cells in fibrosis. Therefore, next to PCKS, we exposed human renal fibroblasts to pirfenidone, galunisertib, and imatinib using a “scar in a jar” model (i.e., in vitro enhancement of collagen matrix deposition, [33]). Lastly, we evaluated the predictive capacity of PCKS by comparing the PCKS responses to pirfenidone, galunisertib, and imatinib treatment with the outcomes of in vivo animal studies and human trials testing the efficacy of these compounds in renal fibrosis (in the discussion).

## 2. Materials and Methods

### 2.1. Ethics Statement

This study was approved by the Medical Ethical Committee of the University Medical Center Groningen (UMCG), according to the Dutch legislation and Code of Conduct for dealing responsibly with human tissue in the context of health research (www.federa.org), forgoing the need of written consent for “further use” of coded-anonymous human tissue. All animal experiments were approved by the Animal Ethics Committee of the University of Groningen, project code DEC6416AA-001 (date of approval 24.09.2014) and DEC6066B (date of approval 16.02.2015) and by the Danish Veterinary and Food Administration, project code 2015-15-0201-00658 (date of approval 03.09.2015).

### 2.2. Chemicals

Three anti-fibrotic compounds were used in this study: pirfenidone (Sigma-Aldrich, Saint Louis, MO, USA), galunisertib (LY2157299; Selleckchem, Munich, Germany), and imatinib (LC Laboratories, Woburn, MA, USA). Stock solutions were prepared in dimethyl sulfoxide (DMSO) and stored at −20 °C. During experiments, stocks were diluted in the culture medium with a final DMSO concentration of ≤0.5%.

### 2.3. Human Material

Macroscopically healthy renal cortical tissue (n = 16) was obtained from tumor nephrectomies, whereas fibrotic renal tissue (n = 9) was obtained from end-stage renal disease (ESRD) nephrectomies or transplantectomies. Table 1 summarizes patient demographics. Renal tissue was kept in ice-cold University of Wisconsin (UW) organ preservation solution, and cold ischemia time from the moment of organ retrieval to PCKS incubation was limited to 2–3 h.

### 2.4. Experimental Animals and Surgical Procedures

As a healthy control group, we used twelve male and female 8–12-week-old C57Bl/6J mice that were bred in the Central Animal Facility of the University Medical Center Groningen (The Netherlands). For the renal fibrosis group, seven male 8–10-week-old C57Bl/6J mice were obtained from Envigo (Horst, The Netherlands) or Janvier Labs (Saint-Berthevin, France). All animals were housed under controlled conditions and had ad libitum access to standard rodent chow and tap water. To induce renal fibrosis, mice were subjected to unilateral ureteral obstruction (UUO) under general anesthesia (isoflurane/O_2_) by double-ligation of the left ureter using 6-0 silk sutures. The right contralateral kidneys were used as controls and were manipulated but not ligated. Mice were sacrificed 3 or 7 days post-UUO. Kidneys from healthy controls and UUO mice were harvested via a terminal procedure performed under 2% isoflurane/O_2_ or sevoflurane anesthesia and kept in ice-cold UW organ preservation solution until slicing.

### 2.5. Preparation, Incubation, and Treatment of Precision-Cut Kidney Slices (PCKS)

Precision-cut kidney slices (PCKS) were prepared using a Krumdieck tissue slicer, as previously described [17,22]. Slices with a wet weight of 4–5 mg and estimated thickness of 250 μm were incubated individually in 12-well culture plates filled with Williams’ Medium E + GlutaMAX (Life Technologies, Carlsbad, CA, USA) that contained 10 μg/mL ciprofloxacin (Sigma-Aldrich, Saint Louis, MO, USA) and 26 mM D-glucose (Sigma-Aldrich, Saint Louis, MO, USA). To investigate the anti-fibrotic effects of the test compounds, slices were treated for 48 h with pirfenidone (2.5 mM), galunisertib (1–10 μM), or imatinib (5–25 μM); slices exposed to DMSO served as a solvent control group. All PCKS were incubated at 37 °C in 80% O_2_/5% CO_2_ atmosphere while gently shaken (90 cycles/min). Medium and added compounds were refreshed at 24 h.

PCKS were collected directly after slicing (0 h) and after 48 h of culture. Biochemical analyses were performed using three pooled slices from the same animal/donor (technical replicates) from 3–5 animals or 5–7 human donors (biological replicates).

### 2.6. PCKS Viability

Viability of the slices was assessed by measuring the adenosine triphosphate (ATP) content using the ATP bioluminescence kit (Roche Diagnostics, Mannheim, Germany), as previously described [34]. The ATP content of each slice was normalized to its total protein content determined using the DC™ Protein Assay (Bio-rad, Veenendaal, The Netherlands) according to the manufacturer’s instructions.

### 2.7. PCKS RNA Isolation and RT-qPCR

Total RNA was extracted from three pooled slices using the RNeasy mini kit (Qiagen, Venlo, the Netherlands), and RNA (1 μg) was reverse transcribed using the Reverse Transcription System (Promega, Leiden, The Netherlands). Complementary DNA was used for quantitative real-time PCR performed with a Viia7 Real-Time PCR system (Applied Biosystems, Bleiswijk, the Netherlands) with specific primers (Table S1) and FastStart Universal SYBR® Green Master (ROX) kit or FastStart Universal Probe Master (ROX) kit (Roche Diagnostics GmbH, Mannheim, Germany). The mRNA expression values were calculated using the 2^−ΔCt^ method [35] with *GAPDH* as reference gene.

### 2.8. PCKS Histology and Immunohistochemistry

PCKS were fixed in 4% buffered formalin, embedded in paraffin, and sectioned at a thickness of 4 μm. Tissue damage and renal fibrosis were assessed by Periodic acid–Schiff (PAS) staining. Additionally, immunohistochemistry was performed for collagen type I and α-SMA. After deparaffinization, antigen retrieval was achieved by treatment with 0.1 M Tris-EDTA (pH 9.0) in the microwave for 15 min. Tissue sections were blocked with 2% rat or human serum in PBS/2% BSA for 10 min and then incubated with the following primary antibodies for 1 h: anti-type I collagen (COLI, 1:400, 1310-01, SouthernBiotech, Birmingham, AL, USA) and anti-alpha smooth muscle actin (α-SMA, 1:400, A2547, Sigma-Aldrich, Saint Louis, MO, USA). Binding of primary antibodies was detected using the appropriate HRP-conjugated secondary and tertiary antibodies (all obtained from Dako, Glostrup, Denmark) and the ImmPact NovaRed kit (Vector, Burlingame, CA, USA), followed by hematoxylin counterstaining. Stained tissue sections were scanned using a Nanozoomer Digital Pathology Scanner (NDP Scan U10074-01, Hamamatsu Photonics K.K., Hamamatsu, Japan). Computer-assisted morphometric image analysis was used to assess the extent of cortico-interstitial type I collagen and α-SMA expression. Whole-slide images were processed with Aperio ImageScope v12.3 (Aperio Technologies, Vista, CA, USA) by applying the Positive Pixel Count V9 algorithm (hue value set to 0) to every image, with 2–3 regions (i.e., kidney slices) quantified per image. Blood vessels positively stained for α-SMA were excluded from the quantitative analysis. Staining intensity was measured as percentages—number of positive and strong positive pixels divided by the total number of pixels—and expressed as relative values to the control group, as previously described [36].

### 2.9. Pro-collagen Iα1 ELISA Measurement in Human PCKS

Culture medium was collected at 48 h from three individual wells for each experimental group. The level of pro-collagen 1α1 protein secreted by human kidney slices into culture medium was measured in duplicate by Human Pro-collagen 1α1 DuoSet ELISA kit (R&D Systems, Abingdon, UK), according to manufacturer’s instructions. The ELISA sensitivity was 31.25–2000 pg/mL.

### 2.10. Cell Culture, Macromolecular Crowding and Treatments

Normal adult primary human renal fibroblasts (HRFs, 061314CA, DV Biologics, Yorba Linda, CA, USA) were propagated in Dulbecco’s modified Eagle medium (DMEM, 12-604F, Lonza, Verviers, Belgium) containing 50 U/L penicillin/streptomycin (pen/strep, 15140122, Thermo Fisher Scientific, Landsmeer, The Netherlands) and 10% fetal bovine serum (FBS, Sigma-Aldrich). Cells were negative for mycoplasm contamination. Once cells reached appropriate confluency, they were trypsinized, reseeded at a density of 10.000 cells/cm^2^, and serum starved for 18 h in DMEM containing 50 U/L pen/strep, 0.5% FBS and 0.17 mM ascorbic acid (A8960, Sigma-Aldrich, Saint Louis, MO, USA). As a next step, in order to enhance extracellular matrix deposition, HRFs were exposed to the macromolecular crowder polyvinylpyrrolidone PVP 40 kDa (PVP-40, 21.5 mg/mL, Sigma-Aldrich, Saint Louis, MO, USA) [37] dissolved in DMEM containing 50 U/L pen/strep, 0.5% FBS and 0.17 mM ascorbic acid, and additionally stimulated with 5 ng/mL TGFβ1 (100-21C, Peprotech, London, UK). At the same time, cells were treated for 48 or 96 h with pirfenidone (0.5–2.5 mM), galunisertib (0.1–5 μM), or imatinib (1–10 μM); cells treated with DMSO were used as control. Medium and compounds were refreshed every 24 h. At least three individual experiments were performed.

### 2.11. RNA Isolation and RT-qPCR (HRFs)

Total RNA was isolated from the cells using the Tissue Total RNA mini kit (Favorgen Biotech Corp., Taiwan). RNA quantity and quality were determined by UV spectrophotometry (NanoDrop Technologies, Wilmington, DE, USA). RNA (1 μg) was reverse transcribed using the RevertAid First Strand cDNA Synthesis kit (Thermo Scientific, Waltham, MA, USA). Real-time quantitative PCR was performed with Taqman gene expression assays (Table S1) and FastStart Universal Probe Master (ROX) kit (Roche Diagnostics GmbH, Mannheim, Germany) and the Viia7 Real-Time PCR system (Applied Biosystems, Bleiswijk, The Netherlands). The mRNA expression values were calculated using the 2^−ΔCt^ method and normalized against *GAPDH*.

### 2.12. Immunocytochemistry (HRFs)

Cells were seeded on 8-well chamber slides at 10.000 cells/cm^2^ and treated with anti-fibrotic compounds as described above. Afterwards, the cell layer was washed twice with PBS and either fixed with 4% paraformaldehyde (Sigma-Aldrich) for 10 min for detection of extracellular type I collagen or fixed with ice-cold methanol/acetone (1:1) for 10 min for detection of α-SMA. Methanol/acetone fixed cells were first dried and then rehydrated with PBS before further use. Non-specific sites were blocked with 2.2% BSA (K1106, Sanquin reagents, Amsterdam, The Netherlands) in PBS for 30 min. Subsequently, cells were incubated with the following primary antibodies (diluted in 2.2% BSA/PBS) for 1 h at RT: mouse anti-human collagen type I (1:1000, ab90395, Abcam, Cambridge, UK) and mouse anti-human α-SMA (1:500, M0851, Dako, Glostrup, Denmark). Bound antibodies were visualized using donkey anti-mouse IgG (H+L) Alexa Fluor 555 (1:1000, A-31570, Thermo Fisher Scientific) for 1 h at RT. Cell nuclei were counterstained with 4′,6-diamidine-2′-phenylindole dihydrochloride (DAPI; 1:5000), and slides were mounted with Citifluor AF1 (AF1-25, Brunschwig Chemie, Amsterdam, the Netherlands). Microphotographs were acquired in a random blind fashion with the use of a Leica DMRA microscope (Leica Microsystems, Rijswijk, The Netherlands). The extent of collagen type I and α-SMA deposition was quantified from at least six regions per well per treatment group (40× magnification) using ImageJ software version 1.52i (http://rsbweb.nih.gov/ij/). Triangle automatic thresholding was applied to discriminate between the fore- and background, and the percentage of collagen or α-SMA positive area in each image was used for statistical analysis.

### 2.13. Statistical Analysis

The results are expressed as mean ± standard error of the mean (SEM). Statistics were performed using GraphPad Prism 6.0 (GraphPad Software Inc.) by unpaired one-tailed Student’s t-test (to compare two groups) or one-way ANOVA followed by Dunnett’s multiple comparisons test (to compare three or more groups). Non-parametric Kruskal–Wallis test followed by Dunn’s multiple comparisons test was used to compare protein levels of collagen type I and α-SMA. A *p*-value lower than 0.05 was considered to represent statistically significant differences.

## 3. Results

### 3.1. Tissue Viability in Response to Ex Vivo Culture and Pharmacological Intervention

**Figure 1:**
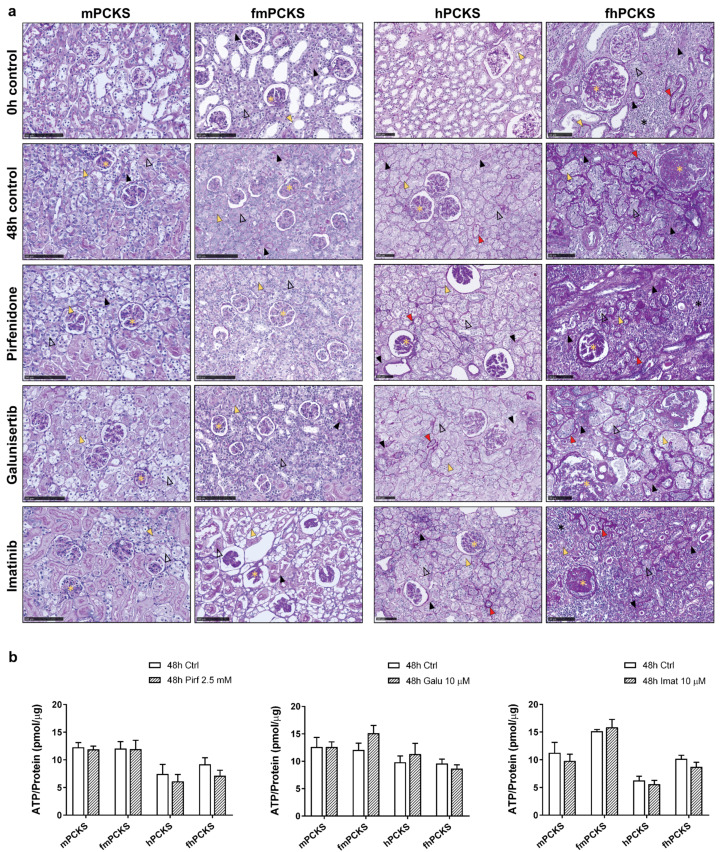
Viability of murine and human PCKS prepared from normal and fibrotic kidneys. PCKS were prepared using Krumdieck tissue slicer and incubated for 48 h with or without compounds—2.5 mM pirfenidone, 10 µM galunisertib, or 10 µM imatinib. Renal tissue viability was assessed histomorphologically using Periodic acid–Schiff (PAS) staining (**a**) and by measuring ATP levels normalized for total protein content at 48 h with and without treatment (**b**). Symbols in (**a**) mark the pathological changes observed in PCKS, such as anucleosis (yellow arrow head), disappearance of the tubular brush border (open black arrow head), thickening of tubular basement membrane (red arrow head), interstitial fibrosis/expansion of interstitial ECM (black arrow head), interstitial inflammation (black star), and glomerular sclerosis of various degree (yellow star). Data in (**b**) are expressed as mean ± SEM, n = 3–5 (murine PCKS) or n = 5–7 (human PCKS). An unpaired two-tailed Student’s t-test was used to test the differences between 48-h Ctrl and 48-h treatment groups; * *p* < 0.05; scale bars are 100 µm. Annotations: mPCKS, murine (healthy control) precision-cut kidney slices; fmPCKS, fibrotic murine slices prepared from obstructed for seven days (7dUUO) kidneys; hPCKS, human (healthy control) kidney slices; fhPCKS, fibrotic human slices prepared from patient diseased renal tissues.

Viability of murine and human PCKS was assessed after 48 h of culture with or without treatment with anti-fibrotic compounds by examining tissue morphology and ATP/protein content, and compared to 0 h PCKS or untreated 48 h PCKS. Prior to culturing, PCKS from healthy murine (mPCKS) and human (hPCKS) kidneys displayed normal cortex architecture with preserved glomerular and tubular structures (Figure 1a). During 48-h culture, mPCKS and hPCKS developed mild tubulointerstitial injury and cellular damage, i.e., pyknosis and anucleosis, as previously described in more detail by Stribos et al. [22]. In turn, PCKS from 7d UUO mouse kidneys (fmPCKS) and fibrotic human (fhPCKS) kidneys already at 0 h showed clear signs of inflammation, tubulointerstitial fibrosis, and glomerular injury, while 48-h culture induced further expansion of interstitial ECM, thickening and wrinkling of tubular basement membranes, disappearance of brush borders, tubular atrophy and glomerular sclerosis. Nevertheless, all slices maintained high ATP levels during incubation (Figure S1a). PCKS were subjected to pharmacological intervention using several compounds with previously reported anti-fibrotic activity—pirfenidone, galunisertib, and imatinib—at concentrations similar to those used in published in vitro studies [38,39,40,41]. Of note, for galunisertib and imatinib, we mainly focused on their effects at 10 µM to stay relatively close to clinically relevant concentrations. Treatment with 2.5 mM pirfenidone, 10 µM galunisertib, or 10 µM imatinib did not cause evident toxicity in PCKS, as assessed by histology (Figure 1a) and ATP content that did not show any significant changes after compound exposure for 48 h (Figure 1b). Lower concentrations of galunisertib and imatinib also did not affect tissue viability, whereas 25 µM imatinib significantly reduced ATP content of fhPCKS by 46% (Figure S1).

### 3.2. Culture-Driven Inflammatory and Fibrogenic State of PCKS

**Figure 2:**
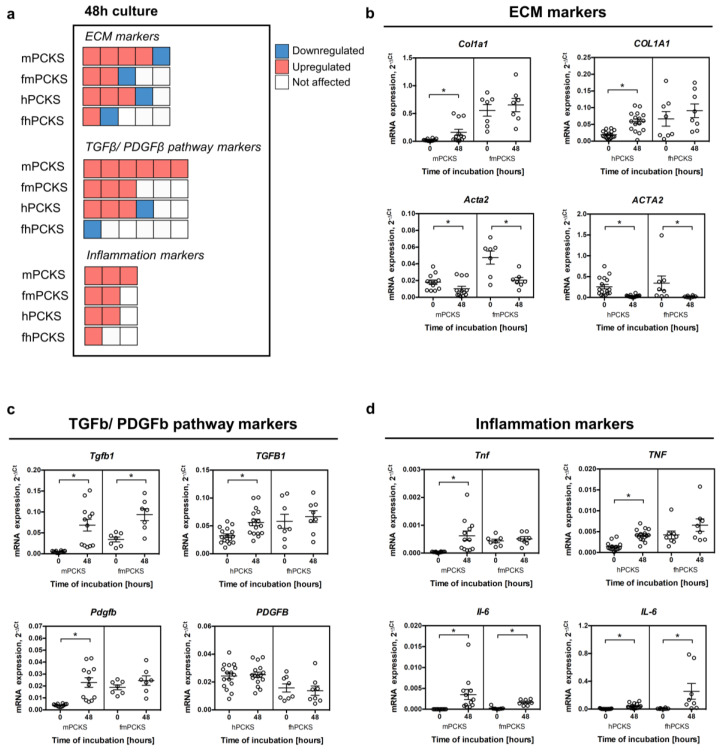
Culture-induced spontaneous fibrogenic and inflammatory responses in PCKS in relation to species and healthy/diseased phenotype. (**a**) Visual summary of changes in the transcription of selected genes in murine and human PCKS prepared from normal (mPCKS and hPCKS) and fibrotic kidneys (7dUUO fmPCKS and fhPCKS) after 48-h culture. Each box represents one gene, while the direction of change is indicated by the color: red, upregulated during 48-h culture; blue, downregulated; and white, not affected by culturing. This diagram only indicates the number of altered genes (without specifying the marker genes) and the direction of change, in the following order: upregulation, downregulation, and no change. Evaluated genes were divided into three categories: extracellular matrix (ECM) markers (*COL1A1, ACTA2, SERPINH1, FN1,* and *PLOD2*), markers of TGFβ and PDGF signaling (*TGFB, TGFBR1, TGFBR2, SERPINE1*, *PDGFB,* and *PDGFRB*), and inflammation markers (*TNF, IL-1B,* and *IL-6*). (**b**–**d**) Examples of regulated transcripts in each category: ECM markers (**b**); markers of TGFβ and PDGF pathways (**c**); and markers of inflammation (**d**). Regulation of rest of the transcripts is illustrated in Figure S2. Data are expressed as mean ± SEM, n = 3–5 (murine PCKS) or n = 5–7 (human PCKS); * *p* < 0.05.

Before elucidating and comparing the impact of the selected drugs in PCKS, we first investigated the effects of 48-h incubation on murine and human slices. To this end, we analyzed the mRNA expression of 14 selected genes related to ECM organization (*COL1A1, ACTA2, SERPINH1, FN1,* and *PLOD2*), TGFβ and PDGF signaling (*TGFB, TGFBR1, TGFBR2, SERPINE1*, *PDGFB,* and *PDGFRB*), and inflammation (*TNF, IL-1B,* and *IL-6*). Figure 2a provides a quick comparison of the magnitude of the culture-induced transcriptional changes across PCKS without detailing marker genes, while Figure 2b–d and Figure S2 provide detailed expression data. In line with previous reports [22,42], preparation and culturing of the slices triggered the early onset of fibrosis and inflammation and activated both the TGFβ and PDGF pathways in PCKS prepared from healthy murine and human kidneys. In particular, 13 out of 14 tested genes were upregulated at 48 h in mPCKS, while hPCKS showed culture-induced upregulation of eight genes, namely *COL1A1, SERPINH1, PLOD2*, *TGFB, SERPINE1*, *PDGFRB, TNF,* and *IL-6* (Figure 2 and Figure S2). Slices prepared from 7dUUO mouse kidneys and human fibrotic kidneys showed a clear fibrotic and inflammatory transcriptional profile prior to culturing: fmPCKS had significantly higher baseline levels of all tested markers, except for *Plod2*, as compared to mPCKS; in turn, fhPCKS showed increased baseline expression of *COL1A1, SERPINE1, TNF,* and *IL-1B*, as compared to hPCKS (Figure S3). Culturing of fibrotic slices sustained their diseased transcriptional profile, as 7 and 2 (out of 14) genes were upregulated at 48 h compared to 0 h in fmPCKS and fhPCKS, respectively. Interestingly, all PCKS showed reduced expression of *ACTA2*, whereas *TGFBR2* was downregulated in human but not murine PCKS. Of note, mPCKS prepared from the contralateral kidneys of the 7dUUO mice had a similar baseline transcriptional profile as mPCKS (Figure S4a). FmPCKS from obstructed kidneys showed diseased gene expression profile already after three days of UUO, and culture further induced the transcription of *Col1a1*, *Serpinh1* and *Fn1* in mPCKS from contralateral and ligated kidneys of UUO mice (Figure S4b,c).

The observed culture-induced effects suggest that murine PCKS develop responses to injury more quickly than human PCKS, as more genes (out of the 14 analyzed genes) were affected by culture of mPCKS at 48 h. Furthermore, PCKS prepared from healthy kidneys (mPCKS and hPCKS) displayed more culture-induced changes in gene expression than PCKS prepared from fibrotic tissues (fmPCKS and fhPCKS). Taken together, 48-h incubation affected all PCKS by inducing and maintaining a fibrogenic and inflammatory environment, making PCKS suitable for studying anti-fibrotic and anti-inflammatory effects of pharmacological interventions.

### 3.3. Ex Vivo Effects of Pirfenidone, Galunisertib and Imatinib in PCKS

**Figure 3:**
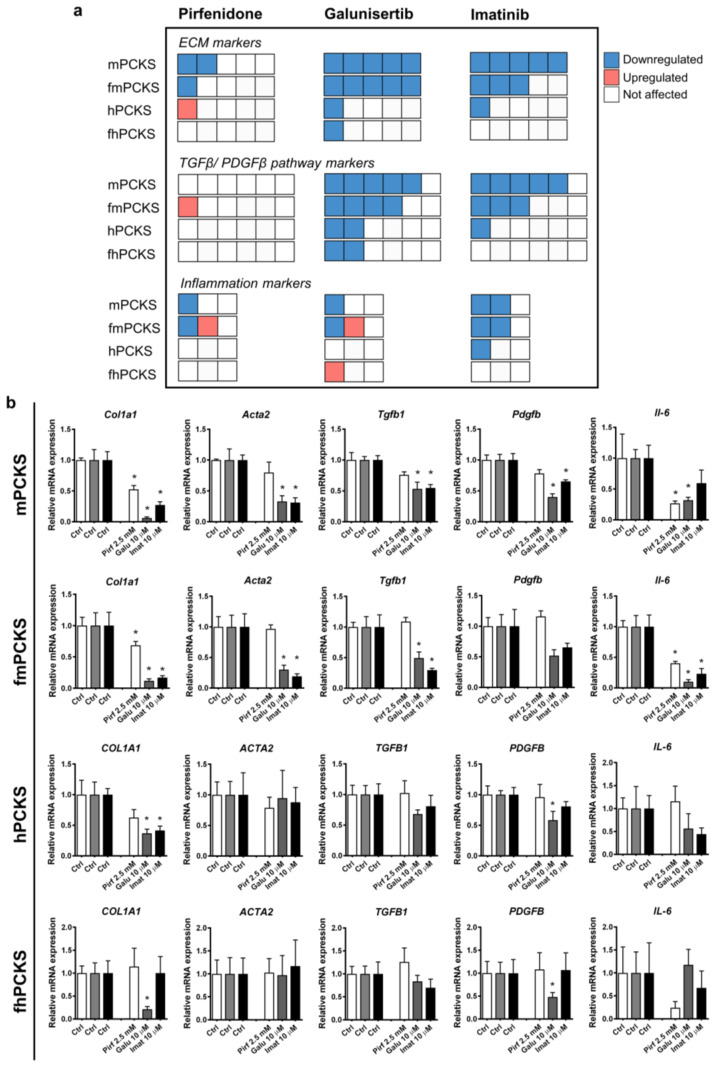
Potency of tested anti-fibrotic compounds to attenuate culture-induced mRNA expression of fibrosis and inflammation markers in PCKS. (**a**) Visual summary of changes in the transcription of selected genes in murine and human PCKS prepared from normal (mPCKS and hPCKS) and fibrotic kidneys (7dUUO fmPCKS and fhPCKS) after 48 h of treatment with pirfenidone 2.5 mM, galunisertib 10 µM, or imatinib 10 µM. Each box represents one gene, while the direction of change is indicated by the color: red, upregulated during 48 h culture; blue, downregulated; and white, not affected by the compound. This diagram only indicates the number of altered genes (without specifying marker genes) and the direction of change, in the following order: downregulation, upregulation, and no change. Evaluated genes were divided in three categories: extracellular matrix (ECM) markers (*COL1A1, ACTA2, SERPINH1, FN1,* and *PLOD2*), markers of TGFβ and PDGF signaling (*TGFB, TGFBR1, TGFBR2, SERPINE1*, *PDGFB,* and *PDGFRB*), and inflammation markers (*TNF, IL-1B,* and *IL-6*). (**b**) Examples of regulated transcripts in each category: ECM markers (*COL1A1* and *ACTA2*), markers of TGFβ and PDGF pathways (*TGFB1* and *PDGFB*), and markers of inflammation (*IL-6*). Regulation of rest of the transcripts is illustrated in Figure S5. Data are expressed as mean ± SEM, n = 3–5 (murine PCKS) or n = 5–7 (human PCKS); * *p* < 0.05.

Next, we evaluated the differences in the effects of pharmacological intervention with pirfenidone, galunisertib, and imatinib on murine and human PCKS prepared from healthy and fibrotic tissues. Figure 3a shows a quick overlook of the transcriptional changes in the expression of the tested ECM markers, markers of TGFβ/PDGF signaling, and inflammation that were observed after 48 h treatment with these compounds. Figure 3b and Figure S5 provide more details regarding the changes in gene expression following treatment. Out of the three tested compounds, galunisertib was the most potent in preventing culture-induced fibrogenic responses in PCKS, and pirfenidone the least.

The effects of pirfenidone at 2.5 mM were limited to significantly inhibited mRNA expression of *Col1a1* and *Il-6* in murine PCKS (healthy and fibrotic). Pirfenidone also inhibited *Fn1* and reduced expression of *Serpinh1* and *Il-1b* by 60%, although not significantly, in mPCKS, but upregulated *Serpine1* and *Il-1b* in fmPCKS. In turn, all human PCKS remained largely unaffected: the only significant impact of pirfenidone on gene expression was the upregulation of PLOD2 in hPCKS (Figure S5). Other effects of pirfenidone in human PCKS were non-significant, including the reduction in expression of *COL1A1* (by 40%), *SERPINH1* (by 20%), and *FN1* (by 30%) in hPCKS, while its effects in fhPCKS were limited to 65% decrease in *IL-1B* and 75% decrease in *IL-6* (Figure 3b and Figure S5).

Murine PCKS displayed greater responsiveness to pharmacological intervention than human PCKS (Figure 3a). Additionally, more genes were affected by the compounds in healthy PCKS compared to those prepared from fibrotic kidneys. For instance, 10 µM galunisertib inhibited mRNA expression of all tested ECM markers in mPCKS and fmPCKS, while it only significantly downregulated *COL1A1* in human slices (Figure 3). In murine PCKS, treatment with galunisertib inhibited *Tgfb1*, *Tgfbr1*, *Serpine1*, *Pdgfb,* and *Pdgfrb* expression, while only *SERPINE1* and *PDGFB* were significantly affected in human PCKS. Galunisertib had a limited effect on the expression of inflammatory markers, as it only inhibited *Il-6* in murine PCKS. Effects of galunisertib in mPCKS were concentration-dependent: only one transcript was downregulated at 1 µM and nine transcripts at 10 µM (Figure S6a). Of note, the inhibitory activity of galunisertib, and not of pirfenidone, on the transcription of ECM markers was also observed in fmPCKS after three days of UUO (Figure S6b).

In the case of imatinib (10 µM), expression of 12 out of 14 genes was decreased in mPCKS after 48 h of treatment. The number of genes significantly affected by imatinib lowered to eight in fmPCKS and three in hPCKS (i.e., *COL1A1*, *PDGFRB,* and *IL-1B*). Compared to galunisertib, treatment with imatinib had a more consistent anti-inflammatory activity. Inhibitory effects of imatinib were observed in mPCKS already at 5 µM, but that was not the case for hPCKS (Figure S6c). Furthermore, imatinib at 10 µM did not significantly affect any of the tested transcripts in fhPCKS (Figure 3a), although it showed minor effect at 25 µM (Figure S6c).

Interestingly, both imatinib and galunisertib halted culture-induced activation of markers related to TGFβ and PDGF pathways in murine PCKS, and these effects were diminished in human PCKS. In turn, pirfenidone only marginally affected TGFβ pathway markers in murine or human PCKS, showing a 25% decrease in mRNA expression of *Tgfb1* in mPCKS and a 30% decrease in *SERPINE1* in both mPCKS and hPCKS, without reaching statistical significance.

**Figure 4:**
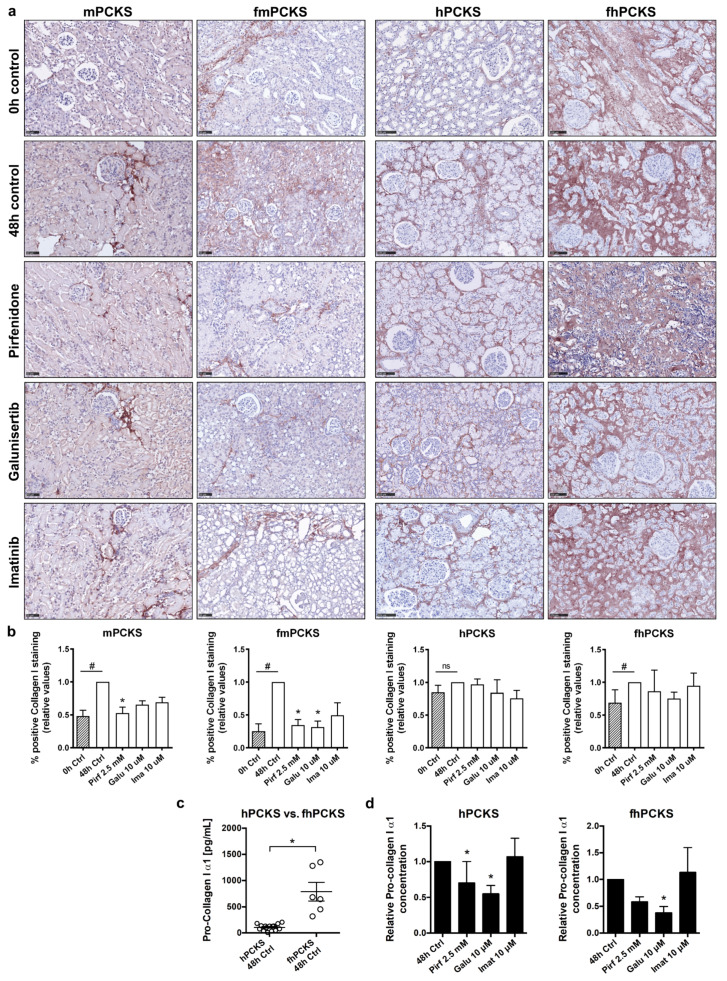
Potency of tested anti-fibrotic compounds to attenuate collagen type I deposition and its de novo biosynthesis in PCKS. (**a**) Representative photomicrographs of immunohistochemistry for collagen type I in murine and human PCKS before (0 h), after culture (48 h), and after treatment with pirfenidone 2.5 mM, galunisertib 10 µM, or imatinib 10 µM. Scale bars are 50 µm (murine PCKS) or 100 µm (human PCKS). (**b**) Computerized quantitative analysis of collagen type I protein expression in PCKS, relative to 48 h control slices. Non-parametric Mann–Whitney test was used to compare collagen levels at 0 and 48 h (control slices), ^#^ *p* < 0.05. Kruskal–Wallis test followed by Dunn’s multiple comparisons test was used to compare treated PCKS with 48 h control slices, * *p* < 0.05. (**c**) Protein levels of procollagen type I (α1), a marker of newly synthesized collagen, secreted by human healthy and fibrotic PCKS during culture, as measured by ELISA in culture supernatants. (**d**) Protein levels of procollagen type I (α1) secreted by human PCKS treated with pirfenidone, galunisertib, or imatinib for 48 h. Data are expressed as mean ± SEM, n = 3–4 (murine PCKS) or n = 3–5 (human PCKS); * *p* < 0.05.

Next, we investigated the changes in protein expression of collagen type I and alpha-smooth muscle actin (α-SMA), in order to assess the ability of the compounds to modulate ECM deposition. Culture for 48 h resulted in a significant increase in accumulation of interstitial type I collagen in mPCKS (*p* = 0.002), fmPCKS (*p* = 0.029), and fhPCKS (*p* = 0.048), but not in hPCKS (*p* = 0.114) (Figure 4a). Immunohistochemistry and morphometric analysis showed that the tested compounds only affected protein expression of collagen type I in murine PCKS (Figure 4a,b). In particular, pirfenidone significantly reduced collagen type I deposition in mPCKS by 48% (*p* = 0.004) and in fmPCKS by 66% (*p* = 0.028). Treatment with 10 µM galunisertib resulted in 34% decrease in collagen type I levels in mPCKS (*p* = 0.077) and 68% decrease in fmPCKS (*p* = 0.022). Of note, lower concentrations of galunisertib showed no effect on ECM deposition in mPCKS (Figure S7a,b). Imatinib (10 µM) also affected interstitial accumulation of collagen type I in murine PCKS, although not significantly, showing 31% reduction in mPCKS (*p* = 0.170) and 51% reduction in fmPCKS (*p* = 0.312).

Given the fact that immunohistochemistry detects total type I collagen fiber content in the slices, we measured the amount of biosynthetic precursor of collagen, pro-collagen I α1, secreted by human PCKS in culture medium to make a distinction between the pre-existing collagen in renal tissue and newly synthesized collagen. Figure 4c demonstrates that fhPCKS secrete approximately seven times more pro-collagen I α1 than hPCKS, further supporting the diseased phenotype of fhPCKS. While the tested compounds did not impact total collagen type I deposition in human PCKS, pirfenidone and galunisertib clearly reduced the amount of soluble pro-collagen I α1 (Figure 4d), illustrating their ability to mitigate de novo biosynthesis of collagen type I. In contrast, 10 µM imatinib failed to show significant effects on both interstitial collagen type I and secreted pro-collagen I in human PCKS. However, when the concentration of imatinib was increased to 25 µM, it significantly reduced levels of collagen type I in mPCKS and hPCKS and decreased soluble pro-collagen type I in hPCKS and fhPCKS (Figure S7c–e).

**Figure 5:**
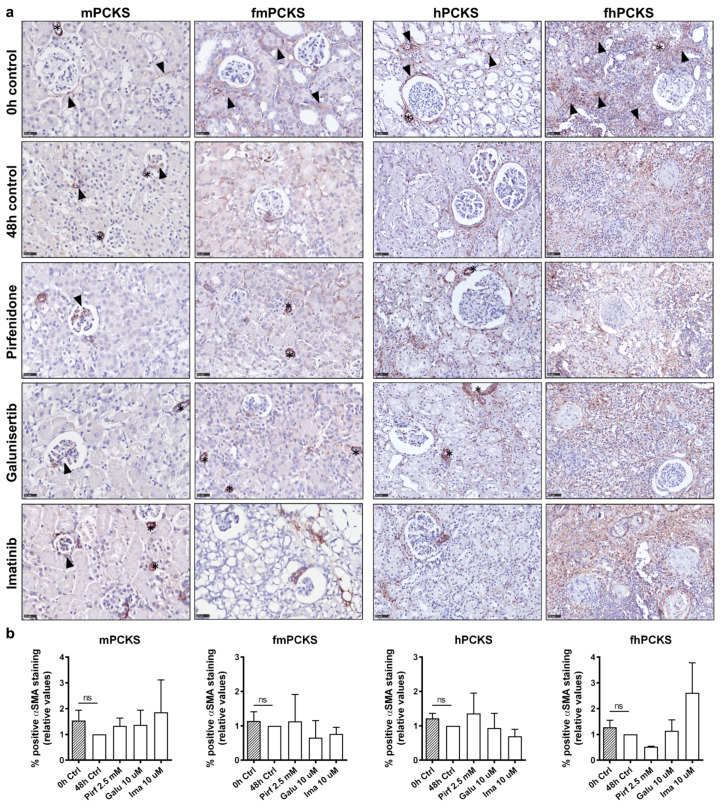
Effect of pirfenidone, galunisertib, and imatinib on interstitial accumulation of alpha-smooth muscle actin (α-SMA) in PCKS. (**a**) Representative photomicrographs of immunohistochemistry for α-SMA in murine and human PCKS before (0 h), after culture (48 h), and after treatment with pirfenidone 2.5 mM, galunisertib 10 µM, or imatinib 10 µM. Scale bars are 25 µm (murine PCKS) or 50 µm (human PCKS). Stars indicate α-SMA-positive blood vessels that were excluded from quantitative analysis. Black arrow heads point α-SMA-positive interstitial and mesangial cells in mPCKS, and show examples of α-SMA expression sites in 0 h control slices. (**b**) Computerized quantitative analysis of α-SMA protein expression in cortico-interstitium in PCKS, relative to 48 h control slices. Non-parametric Mann–Whitney test was used to compare α-SMA levels at 0 and 48 h (control slices), ^#^ *p* < 0.05. Kruskal–Wallis test followed by Dunn’s multiple comparisons test was used to compare treated PCKS with 48 h control slices, * *p* < 0.05. Data are expressed as mean ± SEM, n = 3–4 (murine PCKS) or n = 3–5 (human PCKS).

Figure 5 illustrates the protein expression of α-SMA in murine and human PCKS. Despite the consistently reduced *ACTA2* transcription in PCKS during culture, this effect did not translate to the protein level, as cortico-interstitial expression of α-SMA did not significantly change at 48 h compared to 0 h (Figure 5a,b). Neither of the tested compounds significantly affected protein levels of α-SMA in PCKS after 48 h treatment (Figure 5a,b). Figure S8 shows effects of all tested concentrations of galunisertib and imatinib on α-SMA expression: the only significant change in α-SMA levels was observed in hPCKS upon treatment with imatinib at 5 µM, but not at 10 or 25 µM.

### 3.4. In Vitro Effects of Pirfenidone, Galunisertib and Imatinib Treatment in Primary Human Renal Fibroblasts

To complement and compare the observations in tissue slices with a 2D system, we tested pirfenidone, galunisertib, and imatinib using primary human renal fibroblasts (HRFs). To this end, HRFs were stimulated with the macromolecular crowder PVP-40 and TGFβ to enhance ECM deposition (a culture technique called “scar in a jar”), and exposed to the compounds for 48 h or 96 h. An increased rate of collagen I deposition in the system “scar in a jar” creates a high sensitivity setting to test the effects of anti-fibrotic compounds, while retaining the same genotypical features of cells compared to those not exposed to macromolecular crowder. HRFs were treated with maximally 2.5 mM pirfenidone, 5 µM galunisertib, and 10 µM imatinib, keeping the range of tested concentrations comparable to that used in PCKS experiments. Of note, HRFs were not exposed to 10 µM galunisertib to avoid possible fibroblast toxicity.

**Figure 6:**
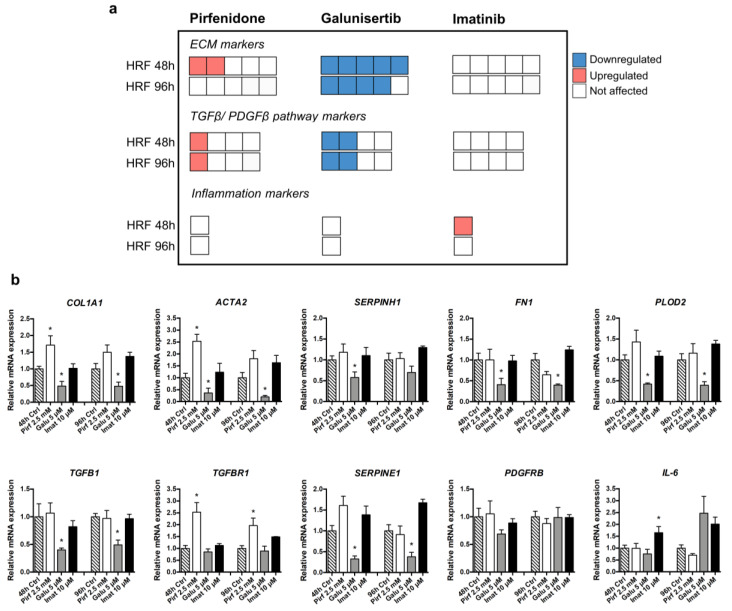
Potency of tested anti-fibrotic compounds to attenuate TGFβ- and PVP40-induced gene expression of fibrosis and inflammation markers in human renal fibroblasts (HRFs). (**a**) Visual summary of changes in the transcription of selected genes in HRFs after 48 h treatment with pirfenidone 2.5 mM, galunisertib 5 µM, or imatinib 10 µM. Each box represents one gene, while the direction of change is indicated by the color: red, upregulated during 48-h culture; blue, downregulated; and white, not affected by the compound. This diagram only indicates the number of altered genes (without specifying marker genes) and the direction of change, in the following order: downregulation, upregulation, and no change. Evaluated genes were divided in three categories: extracellular matrix (ECM) markers (*COL1A1, ACTA2, SERPINH1, FN1,* and *PLOD2*), markers of TGFβ and PDGF signaling (*TGFB, TGFBR1, SERPINE1,* and *PDGFRB*), and inflammation marker *IL-6*. (**b**) Regulation of abovementioned markers in treated HRFs in detail. Regulation of these transcripts by pirfenidone, galunisertib, and imatinib at lower concentrations is illustrated in Figure S9b. Data are expressed as mean ± SEM, n = 3; * *p* < 0.05.

Similar to our PCKS studies, we evaluated changes in the mRNA expression of five ECM markers (*COL1A1*, *ACTA2*, *SERPINH1*, *FN1,* and *PLOD2*), four markers of the TGFβ and PDGF pathways (*TGFB*, *TGFBR1*, *SERPINE1,* and *PDGFRB*), and one inflammatory marker (*IL-6*). Of note, HRFs did not express PDGFB, as it was not detected by RT-qPCR. Time-dependent culture effects in untreated HRFs are shown in Figure S9a. Figure 6 summarizes the transcriptional changes in HRFs following treatment with pirfenidone (2.5 mM), galunisertib (5 µM), and imatinib (10 µM) for 48 and 96 h. Similar to the results obtained with PCKS, galunisertib was found to be the most potent anti-fibrotic compound in HRFs among the three tested drugs. At a concentration of 5 µM, galunisertib inhibited 7 out of 10 of the tested markers at 48 h and 6 out of 10 markers at 96 h (Figure S9b). In contrast, pirfenidone failed to downregulate any of the markers in HRFs at any tested concentration (Figure 6 and Figure S9b). Instead, treatment with pirfenidone resulted in an upregulation of *COL1A1*, *ACTA2*, and *TGFBR1*.

Overall, the number of affected genes suggests that galunisertib possesses greater anti-fibrotic activity in human fibroblasts as compared to human slices. In the case of pirfenidone and imatinib, the lack of an effect in HRFs is in agreement with the observations made in human PCKS.

**Figure 7:**
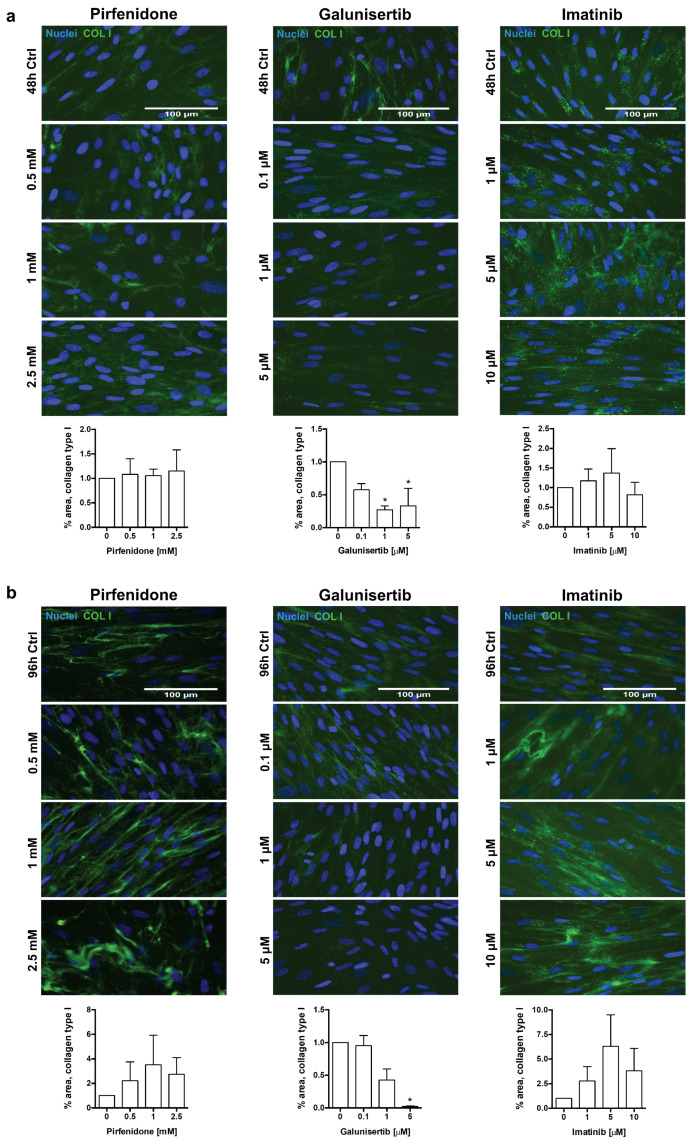
Effect of pirfenidone, galunisertib, and imatinib on extracellular deposition of collagen type I in human renal fibroblasts (HRFs). HRFs were stimulated with macromolecular crowder PVP-40 and TGFβ, and cultured in the presence of pirfenidone (0.5, 1, and 2.5 mM), galunisertib (0.1, 1, and 5 µM), or imatinib (1, 5, and 10 µM) for 48 and 96 h. Representative photomicrographs of immunocytochemistry for extracellular collagen type I fibrils (green) in HRFs treated with compounds for 48 h (**a**) and 96 h (**b**), with respective computerized quantitative analyses. Nuclei (blue); scale bars are 100 µm. Data are expressed as mean ± SEM, n = 3; * *p* < 0.05.

**Figure 8:**
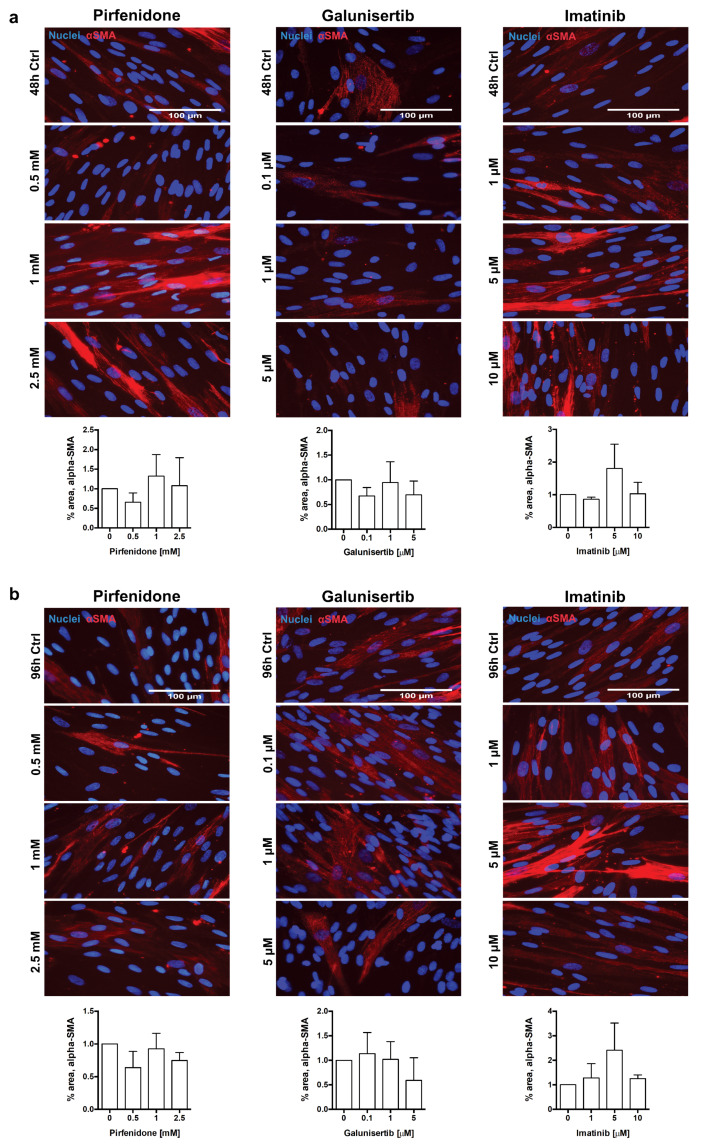
Effect of pirfenidone, galunisertib, and imatinib on accumulation of alpha-smooth muscle actin (α-SMA) in human renal fibroblasts (HRFs). HRFs were stimulated with macromolecular crowder PVP-40 and TGFβ, and cultured in the presence of pirfenidone (0.5, 1, and 2.5 mM), galunisertib (0.1, 1, and 5 µM), or imatinib (1, 5, and 10 µM) for 48 and 96 h. Representative photomicrographs of immunocytochemistry for α-SMA (red) in HRFs treated with compounds for 48 h (**a**) and 96 h (**b**), with respective computerized quantitative analyses. Nuclei (blue); scale bars are 100 µm. Data are expressed as mean ± SEM, n = 3; * *p* < 0.05.

Lastly, we evaluated the impact of treatments on collagen type I and α-SMA deposition in HRFs. Previously, it has been observed that stimulation of human dermal fibroblasts with macromolecular crowders increased accumulation of collagen type I after 48 h of culture [personal communication]. Similarly, stimulation of HRFs with PVP-40 and TGFβ ensured excessive deposition of both collagen type I and α-SMA (data not shown). Immunocytochemistry and morphometric analyses revealed that out of the three tested compounds, only galunisertib reduced collagen type I deposition in HRFs, showing a 67% (*p* = 0.049) decrease in area of fluorescence at 48 h, and a 98% (*p* = 0.019) decrease at 96 h at 5 µM (Figure 7a,b). Similar to PCKS, none of the compounds significantly affected protein levels of α-SMA in HRFs (Figure 8a,b).

## 4. Discussion

Our current understanding of renal fibrosis is for a large part derived from animal studies. In the last decades, a wide variety of models have been established to study renal fibrosis using surgical interventions or the administration of toxic substances to initiate fibrogenesis [1]. Despite great advances in preclinical research, many anti-fibrotics have failed to realize their potential in clinical trials, showing no or limited ability to ameliorate progressive renal disease. Precision-cut kidney slices (PCKS), as an ex vivo translational model, provide a unique opportunity for target validation in patient kidney tissues, which can complement and reinforce in vivo findings in animal models [43]. In this study, we prepared PCKS from murine and human, healthy and fibrotic, renal tissues and established the impact of species and pre-existing pathology on PCKS responses to injury and drug intervention. In particular, we showed that PCKS responded to culturing by undergoing an ECM remodeling (e.g., interstitial accumulation of collagen type I), activation of TGFβ and PDGF pathways and by overexpressing inflammatory genes. Furthermore, murine PCKS responded to injury faster than human PCKS, highlighting species-differences; while culture impacted healthy PCKS to a greater extent than PCKS prepared from fibrotic tissues, suggesting a role of pre-existing inflammatory and fibrotic state in response to injury of the latter. We observed similar trends upon exposure of PCKS to TGFβ and PDGF pathway inhibitors: murine PCKS displayed greater responsiveness to pharmacological intervention than human PCKS (species-differences), while pre-existing pathology of PCKS prepared from fibrotic kidneys limited the effects of compounds as compared to healthy PCKS. Out of the three tested compounds, galunisertib consistently showed the most pronounced anti-fibrotic activity across PCKS, and pirfenidone the least. Lastly, we showed that the anti-fibrotic activity of pirfenidone, galunisertib, and imatinib tested in a 2D system of primary human renal fibroblasts was largely reflected in human PCKS, a 3D ex vivo model.

### 4.1. PCKS Injurious Responses to the Culturing

In this study, we showed that PCKS are suitable for evaluating the potency of therapeutics to directly mitigate fibrosis, since the spontaneous onset of fibrosis in healthy PCKS, triggered by slice preparation and culturing or the diseased state of PCKS obtained from fibrotic kidneys, circumvent the need for an exogenous “hit” to induce fibrogenesis. That is in fact in sharp contrast to many rodent models of renal fibrosis (such as UUO, diabetic nephropathy, lupus nephritis, or chronic allograft nephropathy) that do not allow discriminating whether reduction of established fibrosis by therapeutics was due to amelioration of the underlying disease or the fibrosis itself [31], and to in vitro studies using cell cultures that often require exogenous profibrotic stimuli, such as TGFβ, to augment fibrogenesis.

While responses of PCKS to culturing were previously described [17,20], our study provides for the first time a close comparison of culture-associated changes in PCKS of normal and diseased phenotypes across species. We showed that injury inflicted by slice preparation and sustained by culturing for 48 h affects the transcriptional program of PCKS, dictating faster changes in gene regulation in murine PCKS compared to human PCKS. Independent of the species, PCKS responded to culturing by an increased expression of ECM- and inflammation-related gene markers accompanied by the activation of TGFβ and/or PDGF signaling pathways, in line with previous reports [22,42]. Species differences in injurious responses included unchanged mRNA levels of fibronectin, TGFβ receptor 1, PDGFB ligand and interleukin (IL)-1B in human PCKS as opposed to their increased transcription in mouse PCKS, which might suggest a delayed response of human PCKS, and, therefore, a longer incubation time (beyond 48 h) might be needed to induce changes in these transcripts.

PCKS prepared from (human) fibrotic kidneys have a particular value in basic and translational research, since in clinical practice drug intervention is often initiated in patients at late stages of the disease. We demonstrated that PCKS prepared from fibrotic kidneys are less susceptible to culture-induced changes than PCKS prepared from healthy renal tissues. The pre-existing disease phenotype imposes high baseline expression of ECM- and inflammation-related gene markers, as well as genes involved in TGFβ and PDGF pathways in fibrotic slices, which seems to preclude further induction of their expression by culturing. Consistent with our findings, a study with human liver slices (PCLS) showed that healthy PCLS responded to injury stronger than PCLS prepared from cirrhotic livers: while culture upregulated mRNA levels of collagen type I, fibronectin, HSP47, TGFβ1, and PAI-1 in healthy slices, it failed to do so in cirrhotic liver slices [25]. Nevertheless, we recently showed that culturing of human fibrotic PCKS or cirrhotic PCLS preserves the disease phenotype, while favoring the inflammatory and fibrogenic responses [44].

Myofibroblasts, key activated effector cells during tissue fibrogenesis, are conventionally identified by co-expression of collagen I and α-SMA [45,46]. In contrast to culture-induced increase in collagen type I transcription and protein accumulation, all PCKS showed a dramatic reduction in mRNA levels of α-SMA at 48 h, which in turn was inconsistent with its protein expression. A similar expression pattern of α-SMA was reported in human liver slices [25]. Indeed, it has been shown that, in murine fibrotic lung and kidney, only a minority of collagen-producing cells co-expresses α-SMA, concluding that α-SMA was an inconsistent marker of fibrogenesis [47]. Moreover, in the kidney, expression of α-SMA is not exclusive to myofibroblasts, as it is also present in vascular smooth muscle cells and pericytes [48,49]. Therefore, we advise a cautious assessment of the importance of α-SMA as a fibrosis marker in tissue slices.

### 4.2. PCKS Responses to Pharmacological Treatment in Perspective

**Figure 9:**
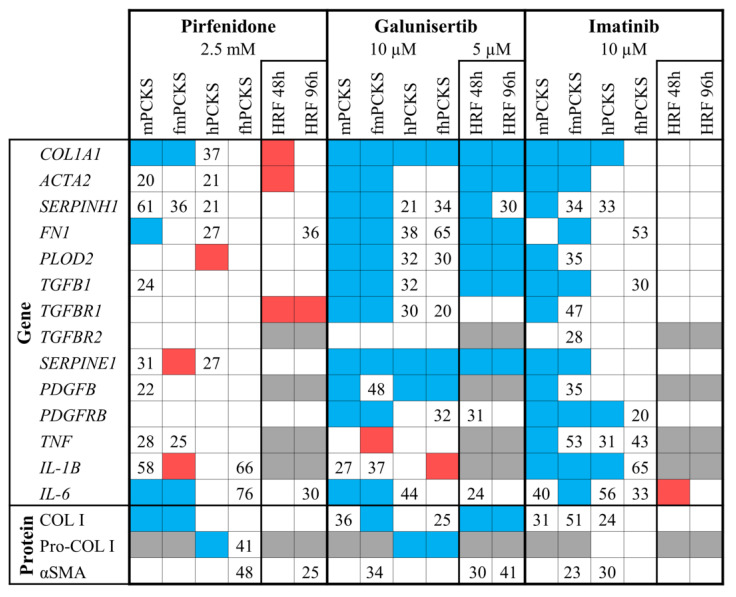
Summary of the observed anti-fibrotic effects of pirfenidone, galunisertib, and imatinib in precision-cut kidney slices (PCKS) and human renal fibroblasts (HRFs). Blue color indicates a significant inhibition, red color shows significant increase, and markers that were not tested in any particular condition are colored in grey. Numbers represent mean percent reduction in gene or protein expression that did not reach statistical significance (only reduction ≥20% is taken into account). Concentration-dependent effects of tested compounds are illustrated in Figure S6 (PCKS) and Figure S9 (HRFs). Annotations: mPCKS, murine (healthy control) precision-cut kidney slices; fmPCKS, fibrotic murine slices prepared from obstructed for seven days (7dUUO) kidneys; hPCKS, human (healthy control) kidney slices; fhPCKS, fibrotic human slices prepared from patient diseased renal tissues.

Figure 9 summarizes the observed ex vivo and in vitro effects of pirfenidone, galunisertib, and imatinib in the present study. A critical step in evaluating the predictive capacity of the PCKS model is to compare the experimental data derived from PCKS with the published in vivo data obtained with the use of established animal models of renal fibrosis. Therefore, in this discussion, we summarize the current knowledge of in vivo responses to pirfenidone, galunisertib, and imatinib in renal fibrosis from animal studies and human trials, and we attempted to draw a parallel between the ex vivo, in vivo and in vitro findings.

#### 4.2.1. Pirfenidone

Discovered in 1976, pirfenidone is a pyridone derivative with anti-fibrotic and anti-inflammatory properties that has been most extensively studied in lung fibrosis. Early studies in bleomycin-induced pulmonary fibrosis model in hamsters indicated that pirfenidone reduced expression of TGFβ and prevented the accumulation of hydroxyproline and procollagen I in lung tissue [50,51]. Although the exact mechanism of action of pirfenidone remains unclear, its anti-TGFβ activity has been implicated to play an important role. Pirfenidone is also known for its anti-fibrotic effects in experimental animal models of renal fibrosis. Table 2 lists the main studies that evaluated the potency of pirfenidone to ameliorate renal fibrosis in vivo. Commonly reported in vivo effects of pirfenidone include: (1) histological improvement of tubulointerstitial fibrosis and glomerulosclerosis (to various degrees); (2) inhibition of cortical collagen type I mRNA and protein expression; and (3) inhibition of TGFβ1 mRNA and protein expression. Several studies also showed that pirfenidone inhibited other fibrogenic factors, such as PAI-1, fibronectin, α-SMA, and MMP2 (Table 2). Three studies demonstrated anti-inflammatory activity of pirfenidone in rodent kidneys (from rats subjected to anti-Thy1 administration or antibodies against glomerular basement membrane to induce glomerulonephritis, or rats subjected to subtotal nephrectomy), illustrated by reduced interstitial inflammatory cell infiltration, reduced accumulation of ED-1 positive macrophages and expression of macrophage markers TNFα and IL-6 [52,53,54]. Ex vivo pirfenidone treatment of murine PCKS from healthy and obstructed kidneys resulted in clear and consistent inhibition of culture-induced collagen type I transcription and protein expression, reflecting in vivo findings. Pirfenidone also mildly reduced mRNA levels of TGFβ1, PAI-1, α-SMA, and HSP47, and significantly inhibited transcription of fibronectin in mPCKS (Figure 9). Similar to animal studies, pirfenidone exerted anti-inflammatory properties in murine PCKS by downregulating gene expression of IL-6 and, to a lesser extent, TNFα.

Still, the promising anti-fibrotic effects observed in animals did not reflect the performance of pirfenidone in patients. In particular, in clinical trials on patients with focal segmental glomerulosclerosis (FSGS) and diabetic kidney disease (DKD), pirfenidone showed conflicting results failing to prevent proteinuria or glomerular filtration rate (GFR) decline [55,56,57,58]. The lack of clinical efficacy was reflected in human PCKS. Ex vivo, pirfenidone did not show strong inhibitory activity in PCKS from normal or fibrotic human kidneys, although it mitigated de novo biosynthesis of collagen type I. Furthermore, pirfenidone failed to affect primary human renal fibroblasts (Figure 9). To our surprise, we were not able to find any other study investigating the effects of pirfenidone in human renal fibroblasts. However, a recent study using human lung fibroblasts revealed that pirfenidone decreased fibroblast proliferation and directly inhibited TFGβ-induced production of collagen type I, α-SMA and HSP47 mRNA, and protein [38,59]. Other in vitro studies showed that pirfenidone suppressed TFGβ-induced effects in rat and human renal tubular epithelial cells [60,61]. Interestingly, Chen et al. [54] suggested that pirfenidone has a protective effect on mitochondria by limiting apoptosis and oxidative stress in proximal tubular cells, a phenomenon that can also be studied in PCKS.

#### 4.2.2. Imatinib

In adult human and murine kidneys, both PDGFRα and PDGFRβ are exclusively expressed in renal mesenchymal cells, i.e., glomerular mesangial cells, vascular smooth-muscle cells, interstitial cortical fibroblasts, and medullary pericytes [12]. During renal fibrosis, all PDGF isoforms and both receptors are upregulated or expressed de novo [32,70]. Indeed, we showed that murine and human PCKS displayed an increased transcription of PDGFB ligand and/or its receptor PDGFRβ during culture, in line with previous studies [17,71]. The clinically available anti-cancer agent imatinib was developed in the late 1990s as a specific inhibitor of bcr-abl protein tyrosine kinase and has been shown to selectively block the PDGF receptor [28]. Due to its ability to attenuate PDGF signaling, imatinib might have therapeutic potential in fibrotic diseases. Table 3 provides an overview of in vivo studies that elucidated the anti-fibrotic activity of imatinib in animal models of renal fibrosis. The most commonly reported in vivo effects of imatinib include: (1) histological improvement of glomerular injury (i.e., reduction of mesangial matrix expansion, glomerular sclerosis, and hypercellularity) and tubulointerstitial damage; (2) inhibition of mRNA and protein expression of PDGFB and its receptors PDGFRα and PDGFRβ; (3) inhibition of TGFβ1 mRNA and protein expression; (4) inhibition of interstitial and/or mesangial expression of α-SMA; and (5) decrease in collagen type IV accumulation, a major glomerular ECM protein. Only two studies demonstrated that imatinib attenuated collagen type I gene expression and protein deposition [72,73], whereas its anti-inflammatory activity (e.g., reduced infiltration of inflammatory cells) was reported by a vast majority of animal studies. It has been shown that the inhibitory effect of imatinib on monocyte/macrophage infiltration is due to abrogation of macrophage colony-stimulating factor (M-CSF)/c-fms signaling [74,75]. Furthermore, the immune modulating properties of imatinib extend to inhibition of B cell and T cell development [76].

The observed effects of imatinib in murine PCKS are largely in line with the aforementioned animal studies. We demonstrated that imatinib attenuated PDGF signaling by inhibiting mRNA expression of PDGFB ligand and its receptor PDGFRβ, and affected transcription of TGFβ1, TGFβ receptor I and PAI-1 in murine PCKS prepared from healthy and 7dUUO kidneys (Figure 9). Furthermore, the anti-fibrotic impact of imatinib was reflected by a consistent reduction in collagen type I gene expression and its interstitial accumulation, as well as by the mitigated transcription of α-SMA, HSP47, and fibronectin. Imatinib exerted strong anti-inflammatory activity in all PCKS, inhibiting expression of TNFα, IL-6, and IL-1B, commonly released by M1 macrophages.

Unfortunately, clinical evidence for the therapeutic efficacy of imatinib in kidney diseases is scarce: despite promising results in animal models of renal fibrosis, only two case reports describe the use of imatinib in patients [77,78]. In one report, imatinib improved renal function and proteinuria in a patient with biopsy-proven membranoproliferative glomerulonephritis (MPGN) and concurrent chronic myeloid leukemia [77]. In the second case, a patient with idiopathic type II cryoglobulinemia with severe kidney involvement showed improved creatinine levels, proteinuria and cryocrit upon imatinib treatment [78]. To date, imatinib has, unfortunately, not been studied in the context of other kidney diseases. Ex vivo effects of imatinib in human PCKS indicate, to some extent, its clinical potential as therapeutic agent for the treatment of renal fibrosis. We demonstrated that imatinib attenuated culture-induced increase in collagen type I transcription and interstitial accumulation in human PCKS from healthy kidneys (Figure 9). Imatinib, at a higher concentration (25 µM), also mitigated de novo synthesis of collagen type I. Furthermore, imatinib blocked PDGFRβ, mildly inhibited TGFβ1 expression, and showed clear anti-inflammatory activity in human PCKS. Nonetheless, imatinib’s capacity to halt established renal fibrosis was very limited, as observed in fhPCKS. Similarly, in liver fibrosis, Neef et al. [79] showed that the beneficial effects of imatinib are limited to the early onset of fibrogenesis and the treatment was rather inefficient in advanced liver injury.

Imatinib did not exert any anti-fibrotic activity in human renal fibroblasts (Figure 9). The majority of animal studies associate the therapeutic efficiency of imatinib with its inhibitory effect on PDGF, however Wang et al. [80] demonstrated that the renoprotective effects of imatinib in UUO rats may result from blocking c-abl kinase, rather than attenuating PDGF signaling. Furthermore, the authors reported that TGFβ selectively activated c-abl in renal fibroblasts, and not in renal mesangial or tubular epithelial cells [80]. In our study, neither of the proposed mechanisms of action of imatinib seemed to be sufficient to attenuate enhanced ECM deposition in human renal fibroblasts. As for anti-inflammatory effects, treatment with imatinib upregulated IL-6 mRNA expression in fibroblasts. It has been previously demonstrated that IL-6 not only promotes pathological inflammatory responses, but can also play a renoprotective role through a mechanism called trans-signaling [81,82].

#### 4.2.3. Galunisertib

Galunisertib is a highly selective inhibitor of TGFβR1 that has been demonstrated to completely inhibit phosphorylation and activation of SMAD2/3 [89]. Although the anti-fibrotic properties of galunisertib have not been widely explored, there is an emerging evidence for its therapeutic efficacy in liver fibrosis [26,27]. To date, the only report showing a potential benefit of galunisertib for the treatment of renal fibrosis in vivo is the study by Ding et al. [90]. This study demonstrated that co-administration of angiotensin II and galunisertib significantly improved renal function in a murine model of hypertensive nephropathy; however, no fibrosis parameters were evaluated.

We demonstrated that, among the three tested compounds, galunisertib showed the most compelling ex vivo anti-fibrotic effect. In murine PCKS from normal and obstructed kidneys, galunisertib consistently inhibited culture-induced collagen type I mRNA expression and interstitial accumulation (Figure 9). Galunisertib also reduced the mRNA expression of α-SMA, HSP47, PLOD2, and fibronectin, suggesting a profound effect on ECM. The impact of galunisertib on ECM remodeling is in line with our previous study demonstrating that galunisertib inhibited the transcription of several collagens in murine PCKS, including collagen type III, V and VI, along with 22 other genes involved in ECM regulation [91]. Furthermore, galunisertib consistently attenuated TGFβ and PDGF signaling (already at 5 µM) and exerted moderate anti-inflammatory activity. We previously reported that besides inhibiting TGFβ1, TGFβRI and PAI-1 transcription, galunisertib blocks phosphorylation of SMAD2 and is selective towards canonical TGFβRI/ALK5 signaling in murine PCKS [91]. The strong anti-fibrotic effects of galunisertib observed in murine PCKS may predict its performance in animal models of renal fibrosis.

Remarkably, galunisertib showed promising anti-fibrotic activity in the early onset of fibrogenesis and in established fibrosis. In human PCKS, galunisertib consistently inhibited collagen type I transcription and de novo synthesis, and showed moderate reduction of HSP47, PLOD2, and fibronectin mRNA levels (Figure 9). In addition, galunisertib, attenuated TGFβ and PDGF signaling in human PCKS prepared from healthy and fibrotic kidneys, as illustrated be a reduction in PAI-1 and PDGFB transcription. Previously, Luangmonkong et al. [25] showed that 10 µM galunisertib blocked phosphorylation of SMAD2 and inhibited transcription of TGFβ1 and collagen type I in human liver slices prepared from healthy and cirrhotic livers, supporting the notion that the anti-fibrotic effects of galunisertib extend to advanced tissue injury. Furthermore, in contrast to pirfenidone and imatinib, treatment with galunisertib effectively mitigated TGFβ-induced ECM deposition in the “scar in a jar” model. Galunisertib displayed strong inhibitory effects on collagen type I and α-SMA transcription in human renal fibroblasts, which also translated to the protein level, reduced the expression of genes encoding HSP47, PLOD2 and fibronectin, and attenuated TGFβ signaling. We have to note that the anti-fibrotic activity of galunisertib in primary human fibroblasts was more pronounced than in human PCKS. There are a few possibilities to consider: (1) cell culture experiments are often narrowed down to elucidating treatment effects in one cell type, therefore some of these effects might be “masked” in a multicellular model as PCKS; and (2) fibroblast stimulation with exogenous TGFβ might result in biased assessment of anti-fibrotic drug activity, especially when testing TGFβ inhibitors. Taken together, marked ex vivo therapeutic efficacy of galunisertib in human renal tissue suggests that it may be a promising treatment for progressive renal fibrosis in patients.

## 5. Conclusions

In summary, our study demonstrates that PCKS is a powerful predictive tool for ex vivo screening of putative drugs for renal fibrosis. We showed that the anti-fibrotic activity of compounds targeting TGFβ and PDGF pathways in murine PCKS corresponded to their therapeutic efficacy in vivo. Considering the poor translation from animal studies to the clinic, the use of human PCKS provides an excellent opportunity for target and therapy validation directly in healthy as well as patient kidney tissue. This does not forego the advantages of cell cultures, murine PCKS, and animal studies in providing insights into the mechanism of action of a drug. Overall, the PCKS model appears to be an important addition to the available toolkit used to advance preclinical drug discovery.

## Figures and Tables

**Table 1:** Patient demographics.

Parameter	Healthy Renal Tissue (n = 16)	Fibrotic Renal Tissue (n = 9)
Gender (% male)	56	33
Age (in years)	61 ± 14	41 ± 12
Serum creatinine before nephrectomy (umol/L)	76.5 ± 24.2	420.0 ± 407.6
eGFR before nephrectomy (ml/min/1.73 m^2^) *	86.6 ± 19.3	NA

* Calculated using the Modification of Diet in Renal Disease (MDRD) formula. Values are presented as the mean ± standard deviation or otherwise if indicated. Abbreviations: eGFR, estimated glomerular filtration rate; NA, not available.

**Table 2:** Studies reporting in vivo effects of pirfenidone.

Model of Renal Fibrosis	Intervention	Main Reported Effects on Renal Fibrosis	Ref
Mesangial proliferative (anti-Thy1) glomerulonephritis (Wistar rats)	500 mg/kg; daily, mixed in the food, for 42 days	- Pirfenidone reduced mesangial matrix expansion and interstitial fibrosis (evaluated by HE, PAS and PAM staining); - reduced protein expression of **type I collagen** and **α-SMA** in glomeruli, affecting mesangial cell transdifferentiation to myofibroblasts (evaluated by immunofluorescence); - suppressed accumulation of ED-1 positive macrophages; - attenuated interstitial inflammatory cell infiltration.	[52]
Streptozotocin-induced diabetes (Wistar rats)	200 mg/kg, daily, mixed in drinking water, for 4 weeks (treatment started 2 weeks after ablation)	- Pirfenidone attenuated fibrotic changes in the kidneys of diabetic rats; - reduced **collagen** deposition (evaluated by % Sirius red staining); - decreased renal and plasma concentration of **fibronectin**;	[62]
Salt-depletion model with ciclosporin-induced renal fibrosis (Sprague-Dawley rats)	salt-depleted diet for 7 days prior to the 28-day treatment with PFD at 250 mg/kg alone or in combination with CSA; daily treatment; 0.5% PFD mixed in the low-salt diet	- Pirfenidone attenuated CSA-induced tubulointerstitial fibrosis (by 48%) and tubular atrophy (histological score on PAS stained sections); - reduced renal cortical **TGFβ1** mRNA and protein expression in CSA-treated rats; - reduced CSA-induced mRNA expression of **PAI-1** and biglycan.	[63]
Salt-depletion model with ciclosporin-induced renal fibrosis (Sprague-Dawley rats)	salt-depleted diet for 7 days prior to the 28-day treatment with PFD at 250 mg/kg, 500 mg/kg or 750 mg/kg in combination with CSA or tacrolimus (FK). Daily treatment; PFD was mixed in the low-salt diet.	- Pirfenidone did not significantly affect CSA-induced mRNA expression of **TGFβ1**; - reduced CSA-induced mRNA expression of type III collagen and TIMP-1; - restored CSA-inhibited mRNA expression of MMP2, and showed no effect on MMP9; - this model failed to display histological tubulointerstitial fibrosis with the addition of CSA or FK, so any effect of pirfenidone on structural parameters could not be seen.	[64]
Diabetic nephropathy (C57BL6 *db/db* mice)	25 mg/day; 0.5% PFD mixed in the food; for 4 weeks total from week 17 to week 21	- Pirfenidone reduced mesangial matrix expansion; - reduced the diabetic stimulation of renal **type I collagen**, type IV collagen, and **fibronectin** gene expression to control levels.	[65]
Anti-glomerular basement membrane glomerulonephritis (Wistar rats)	500 mg/kg; daily by oral gavage, for 8 weeks	- Pirfenidone attenuated glomerular segmental sclerosis and tubular degeneration (evaluated by histological score on PAS); only minor interstitial fibrosis was observed; - reduced podocyte injury; - reduced mRNA expression of **type I collagen**; - showed increased number of ED-1 positive glomerular macrophages, but reduced number of ED-1 positive interstitial cells.	[53]
Focal segmental glomerulosclerosis (FGS/Kist mice)	50 mg/kg; daily, 0.5% PFD mixed in the food; for up to 3 months	- Pirfenidone attenuated the development of glomerulosclerosis and tubulointerstitial fibrosis after 3 months of treatment (histological score); - reduced mRNA expression of **TGFβ1** after 2 months; - showed no clear effect on mRNA expression of TIMP-1 and MMP3.	[66]
Subtotal nephrectomy (rats)	500 mg/kg; daily, mixed in the food, for 12 weeks	- Pirfenidone reduced collagen accumulation (detected by hydroxyproline content) in the cortex of the remnant kidney; - reduced mRNA expression of type IV and **type I collagen** - suppressed the increased mRNA expression of **TGFβ1**.	[67]
Subtotal nephrectomy (Wistar rats)	700 mg/kg; daily, 1% PFD mixed in the food, for 8 weeks	- Pirfenidone attenuated interstitial fibrosis in the cortex (evaluated by Azan staining), although did not show prominent effects on glomerular and tubular degeneration; - reduced mRNA expression of **TGFβ1** and protein expression of **fibronectin**; - had little effect on the expression of **α-SMA** (i.e., myofibroblast differentiation); - showed no effect on infiltration of inflammatory cells in the cortical interstitium.	[68]
Subtotal nephrectomy (Sprague-Dawley rats)	500 mg/kg; once a day by oral gavage for 12 weeks	- Pirfenidone attenuated interstitial fibrosis in the cortex and reduced the scores of glomerulosclerosis (evaluated by PAS and Masson staining); - decreased protein expression of **TGFβ1**, CTGF, **α-SMA**, **fibronectin**, and fibroblast-specific protein-1 (FSP-1); - inhibited mRNA and protein expression of chemokines MCP-1 and MIP-1α, as well as **TNFα**, **IL-6** and iNOS expressed by M1 macrophages, and CD206 and CD86 expressed by M2 macrophages; - decreased the infiltration of inflammatory cells in the cortical interstitium; - decreased tubulointerstitial and glomerular macrophage infiltration.	[54]
UUO model (Sprague-Dawley rats)	500 mg/kg; daily, mixed in the food, for up to 21 days	- Pirfenidone reduced **collagen** accumulation (detected by hydroxyproline content) at 14 and 21 days of UUO; - reduced type IV collagen mRNA expression at 14 and 21 days of UUO (and not after 7 days); - reduced **type I collagen** mRNA expression at 21 days UUO; - inhibited mRNA expression of MMP2 at 14 and 21 days; - inhibited increases in **TGFβ1** mRNA after 7 days; - attenuated fibrotic lesions; - pirfenidone does not reverse the increased collagen accumulation in the acute phase of UUO (i.e., 3 and 7 days).	[69]

HE, hematoxylin-eosin; PAS, periodic acid- Schiff; PAM, periodic acid-silver methenamine; PFD, pirfenidone; CSA, cyclosporine; FK, tacrolimus; FGS, focal glomerular sclerosis; UUO, unilateral ureteral obstruction.

**Table 3:** Studies reporting in vivo effects of imatinib.

Model of Renal Fibrosis	Intervention	Main Reported Effects on Renal Fibrosis	Ref
Mesangial proliferative (anti-Thy-1) glomerulonephritis (Wistar rats)	50 mg/kg; daily by intraperitoneal injections, for 6 days	- Imatinib ameliorated glomerular hypercellularity; - reduced mesangial cell proliferation (ED-1 and BrdU double IHC); - reduced activation of mesangial cells (**α-SMA** IHC) and type IV collagen deposition; - no effect on glomerular macrophage infiltration and proliferation.	[83]
Chronic allograft nephropathy (allografts from Dark Agouti to Wistar-Furth rats)	10 mg/kg; daily by oral gavage for 5 or 90 days	- Imatinib inhibited the development of fibrosis associated with chronic allograft nephropathy (Masson trichrome staining); - reduced protein expression of ligands PDGFA and **PDGFB**, and receptors PDGFRα and **PDGFRβ**; - inhibited infiltration of macrophages and CD4+ T cells into the grafts.	[84]
Diabetic nephropathy (C57BL6 apolipoprotein E–knockout mice)	10 mg/kg; daily by oral gavage for 20 weeks	- Imatinib reduced glomerular injury (attenuated increased glomerular size and mesangial area) and tubulointerstitial area; - prevented the increase in **α-SMA**–positive cell expression in the glomeruli and in the tubulointerstitium in diabetic mice; - reduced accumulation of **collagen type I** and type IV; - reduced expression of Ki-67–positive cells; - reduced **PDGFB** protein and gene expression; - reduced **PDGFRβ** gene expression; - reduced **TGFβ1** protein expression, but not gene expression; - reduced CTGF protein and gene expression; - decreased macrophage infiltration.	[72]
Lupus nephritis (MRL/*lpr* mice)	10 mg/kg or 50 mg/kg; daily by oral gavage 4 times per week up to 8 weeks	Imatinib at 50 mg/kg: - ameliorated glomerulonephritis (reduced proliferation of glomerular cells, infiltrating inflammatory cells and reduced mesangial matrix); - reduced proportional area of glomeruli staining positive for **PDGFRβ**; - reduced expression mRNA levels of **PDGFRβ** and **TGFβ1**. - Treatment with 10 mg/kg imatinib did not affect PDGF signaling, and thus failed to ameliorate the nephritis.	[85]
Lupus nephritis (New Zealand Black/White (NZB/W) F1 hybrid mice)	50 mg/kg; twice a day by oral gavage for 3 months (or longer for survival studies)	- Imatinib limited glomerular hypercellularity, IgG deposits, and tubulointerstitial damage; - reduced interstitial expression of **α-SMA** in fibroblasts; - reduced mRNA expression of **TGFβ1;** - reduced infiltrates of monocytes/macrophages.	[86]
Cryoglobulinemic MPGN (TSLP-transgenic mice)	50 mg/kg; daily by intraperitoneal injection for 2, 4 or 8 weeks	- Imatinib decreased accumulation of collagen IV; - reduced mesangial cell activation (measured by % GTA occupied by **α-SMA**–positive cells); - parenchymal injury and fibrosis were attenuated in a time-dependent manner. - reduced the infiltration of inflammatory cells; - inhibited B cell development;	[87]
Anti-glomerular basement nephritis (Wistar-Kyoto rats)	50 mg/kg; intraperitoneal injection, from one day before up to 13 days (early treatment) or from day 7 to 20 (late treatment)	- Imatinib reduced glomerular fibrin deposition; - ameliorated glomerular injury (reduced crescents formation and necrosis evaluated by morphological analysis); - attenuated the **PDGFRβ** mRNA upregulation; - attenuated the c-fms and M-CSF mRNA expression; - reduced glomerular macrophage accumulation, along with an inhibition of **IL-1b** or MCP-1 expression.	[75]
Anti-glomerular basement nephritis (Wistar-Kyoto rats)	25 mg/kg; daily by intraperitoneal injection, from day 7 to day 49 (long-term treatment) or from day 7 to 13 (short-term treatment)	- Imatinib reduced glomerulosclerosis and improved tubulointerstitial damage in rats with NTS nephritis (evaluated by histological analysis); - decreased **collagen type I** gene expression; - reduced **TGFβ1** gene and protein expression.	[73]
UUO model (Sprague-Dawley rats)	dose-accelerating schedule for 7 days (days 1-2: 50 mg/kg; days 3-4: 100 mg/kg; days 5–7: 150 mg/kg), once daily by intraperitoneal injection	- Imatinib blocked TGFβ-stimulated c-abl activity in fibroblasts in obstructed kidneys; - did not block increased **PDGFB** expression, neither affected increased **PAI-1** levels; - reduced proliferation of vimentin-expressing fibroblasts in obstructed kidneys, although induction of **α-SMA** in fibroblasts was not mitigated; - reduced fibrogenesis, as indicated by reduced accumulation of collagen type III and IV and fibronectin in the renal interstitium; - did not reduce interstitial macrophage infiltration.	[80]
UUO model (C57BL6 wild-type or Coll-GFP mice)	50 mg/kg; 2h before the surgery, then twice a day until sacrifice on day 4 or 14	- Imatinib decreased PDGFRα and **PDGFRβ** phosphorylation in pericytes in UUO kidneys; - decreased the expanded population of myofibroblasts by inhibiting cell proliferation; - attenuated fibrosis development in UUO kidneys (measured by % Sirius red area); - attenuated macrophage infiltration.	[88]
Unilateral IRI model (C57BL6 wild-type or Coll-GFP mice)	50 mg/kg; 2h before the surgery, then twice a day until sacrifice on day 4 or 14	- Imatinib inhibited pericyte activation and expansion of myofibroblasts in IRI kidneys on days 4 and 14 post IRI; - attenuated interstitial fibrosis on day 14 post IRI (measured by Sirius red staining).	[88]

IHC, immunohistochemistry; IgG, immunoglobulin G; MPGN, membranoproliferative glomerulonephritis; TSLP, thymic stromal lymphopoietin; GTA, glomerular tuft area; NTS, nephrotoxic serum; UUO, unilateral ureteral obstruction; IRI, ischemia/reperfusion injury.

## Supplementary Materials

The following are available online at https://www.mdpi.com/1999-4923/12/5/459/s1, Table S1: List of primers used for qRT-PCR, Figure S1: Viability of PCKS treated with increasing concentrations of galunisertib and imatinib, Figure S2: Complementary information on culture-induced spontaneous fibrogenic and inflammatory responses in murine and human PCKS, Figure S3: Baseline transcriptional profiles of murine and human PCKS showcasing the pre-existing diseased state of PCKS prepared from fibrotic kidneys prior culturing, Figure S4: Baseline and culture-driven transcriptional regulation of fibrosis and inflammation markers in murine PCKS from healthy and UUO mice, Figure S5: Complementary information on potency of tested anti-fibrotic compounds to attenuate culture-induced expression of fibrosis and inflammation markers in PCKS, Figure S6: Visual summary of concentration-dependent effects of pirfenidone, galunisertib, and imatinib in PCKS, Figure S7: Concentration-dependent effects of galunisertib and imatinib on collagen type I deposition and its de novo biosynthesis in PCKS, Figure S8: Concentration-dependent effects of galunisertib and imatinib on accumulation of alpha-smooth muscle actin (α-SMA) in PCKS, Figure S9: Transcriptional changes in human renal fibroblasts (HRFs) during culture and concentration-dependent anti-fibrotic effects of tested compounds.
