# Supplementary material for: Predictive Value of Precision-Cut Kidney Slices as an Ex Vivo Screening Platform for Therapeutics in Human Renal Fibrosis
Source: Pharmaceutics. 2020 May 18;12(5):459. doi: 10.3390/pharmaceutics12050459 (PMC7285118; doi:10.3390/pharmaceutics12050459)
Supplement: Supplementary file 1 [file pharmaceutics-12-00459-s001.pdf]

pharmaceutics

Supplementary Materials: Predictive Value of Precision-Cut Kidney Slices as an Ex Vivo Screening Platform for Therapeutics in Human Renal Fibrosis

Emilia Bigaeva, Nataly Puerta Cavanzo, Elisabeth G.D. Stribos, Amos J. de Jong, Carin Biel, Henricus A.M. Mutsaers, Michael S. Jensen, Rikke Nørregaard, Anna M. Leliveld, Igle J. de Jong, Jan-Luuk Hillebrands, Harry van Goor, Miriam Boersema, Ruud A. Bank and Peter Olinga *

Table S1. List of primers used for qRT-PCR.

	Gene symbol	Forward sequence	Reverse sequence
Mouse	Col1a1	TGACTGGAAGAGCGGAGAGT	ATCCATCGGTCATGCTCTCT
	Acta2	CTCTCTTCCAGCCATCTTTCAT	TATAGGTGGTTTCGTGGATGC
	Serpinh1	GAAGGCTGTCGCCATCTC	CCCAGTCCTGCCAGATGT
	Fn1	GAGCTATCCATTTCACCTTCAGA	TTGTTCGTAGACACTGGAGAC
	Plod2	CAGAGGAGAACCTAAGTCAAGCA	ATCCTGACGGCAGAAATCC
	Tgfb1	CCGAATGTCTGACGTATTGAAGA	GCGGACTACTATGCTAAAGAGG
	Tgfbr1	AAATTGCTCGACGCTGTTCT	GGTACAAGATCATAATAAGGCAACTG
	Tgfbr2	CCATGGCTCTGGTACTCTGG	ATGGGGGCTCGTAATCCTT
	Serpine1	AGGATCGAGGTAAACGAGAGC	GCGGGCTGAGATGACAAA
	Pdgfb	CGGCCTGTGACTAGAAGTCC	GAGCTTGAGGCGTCTTGG
	Pdgfrb	TCAAGCTGCAGGTCAATGTC	CCATTGGCAGGGTGACTC
	Tnf	CTGTAGCCCACGTCGTAGC	TTGAGATCCATGCCGTTG
	Il-1b	CTCCACCTCAATGGACAGAA	GCCGTCTTTCATTACACAGG
	Il-6	TGATGCTGGTGACAACCACGGC	TAAGCCTCCGACTTGTGAAGTGGTA
	Gapdh	TGCACCACCAACTGCTTAG	GGATGCAGGGATGATGTTC
Human	**Gene symbol**	**Taqman assay ID**	
	COL1A1	Hs00164004_m1	
	ACTA2	Hs00426835_m1	
	SERPINH1	Hs01060397_g1	
	FN1	Hs01549976_m1	
	PLOD2	Hs01118190_m1	
	TGFB1	Hs00998133_m1	
	TGFBR1	Hs00610320_m1	
	TGFBR2	Hs00234253_m1	
	SERPINE1	Hs01126606_m1	
	PDGFB	Hs00966522_m1	
	PDGFRB	Hs01019589_m1	
	TNF	Hs01113624_g1	
	IL-1B	Hs01555410_m1	
	IL-6	Hs00985639_m1	
	GAPDH	Hs02758991_g1	

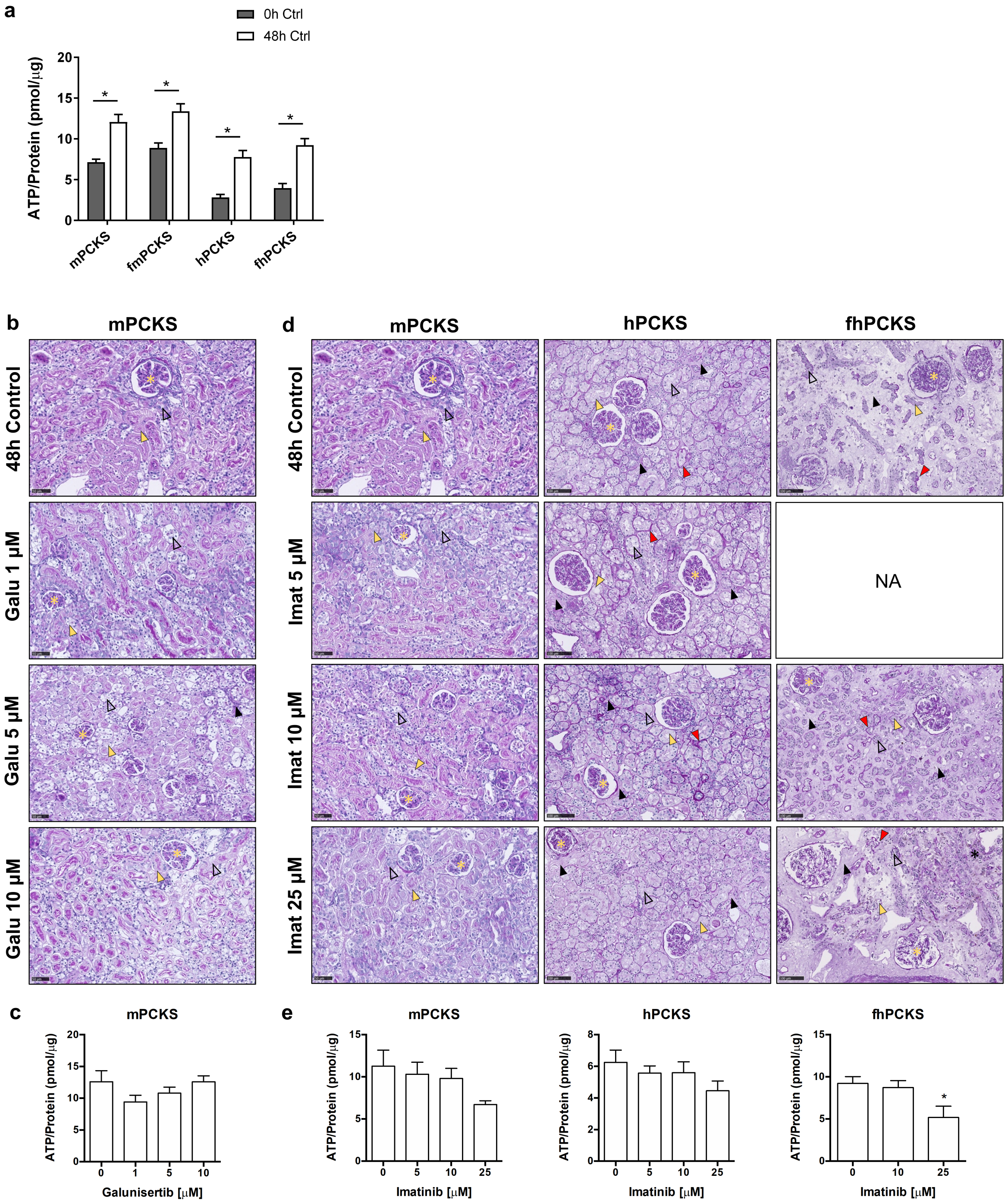

Figure S1. Viability of PCKS during culture and treated with increasing concentrations of galunisertib and imatinib. (a) Viability of murine and human PCKS prepared from healthy or fibrotic kidneys during 48h incubation as measured by ATP levels normalized for total protein content. Murine healthy control PCKS were treated with 1-10 μM galunisertib for 48h (b-c); murine and human PCKS were treated with 5-25 μM imatinib (d-e). Renal tissue viability after treatment was assessed histomorphologically using Periodic acid–Schiff (PAS) staining (b, d) and by measuring ATP levels normalized for total protein content (c, e). Symbols in (b and d) mark the pathological changes observed in PCKS, such as anucleosis (yellow arrow head), disappearance of the tubular brush border (open black arrow head), thickening of tubular basement membrane (red arrow head), interstitial fibrosis/ expansion of interstitial ECM (black arrow head), interstitial inflammation (black star) and glomerular sclerosis of various degree (yellow star). Data in (a, c and e) are expressed as mean ± SEM, n=3-5 (murine PCKS) or n=5-7 (human PCKS); **p* < 0.05; scale bars are 50 μm (murine PCKS) or 100 μm (human PCKS).

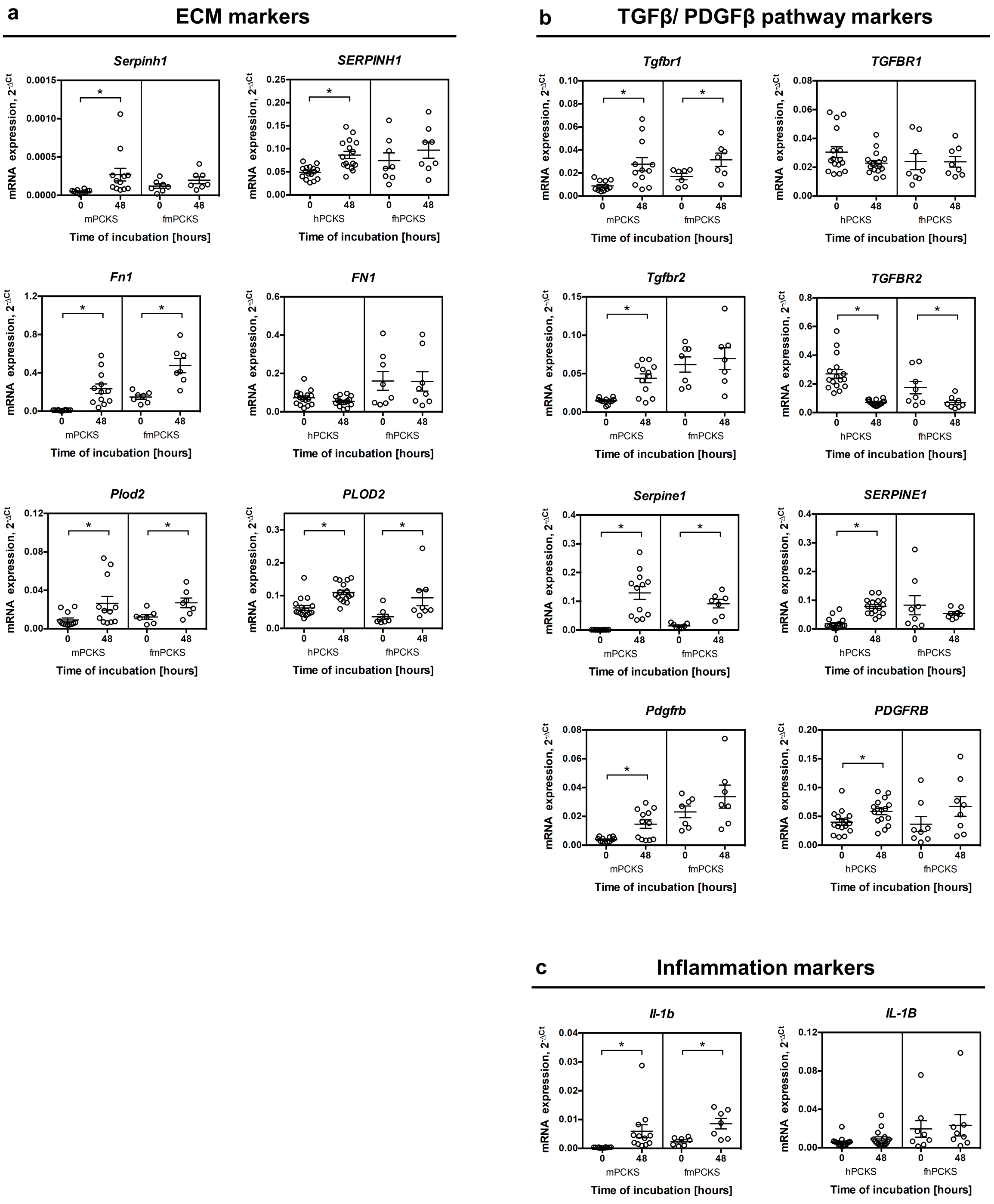

Figure S2. Complementary information on culture-induced spontaneous fibrogenic and inflammatory responses in murine and human PCKS. Complementary information for Figure 2, as it illustrates the regulation of the rest of tested markers: ECM markers (a), markers of TGFβ and PDGF pathways (b), and markers of inflammation (c). Data are expressed as mean ± SEM, n=3-5 (murine PCKS) or n=5-7 (human PCKS); *$p < 0.05$.

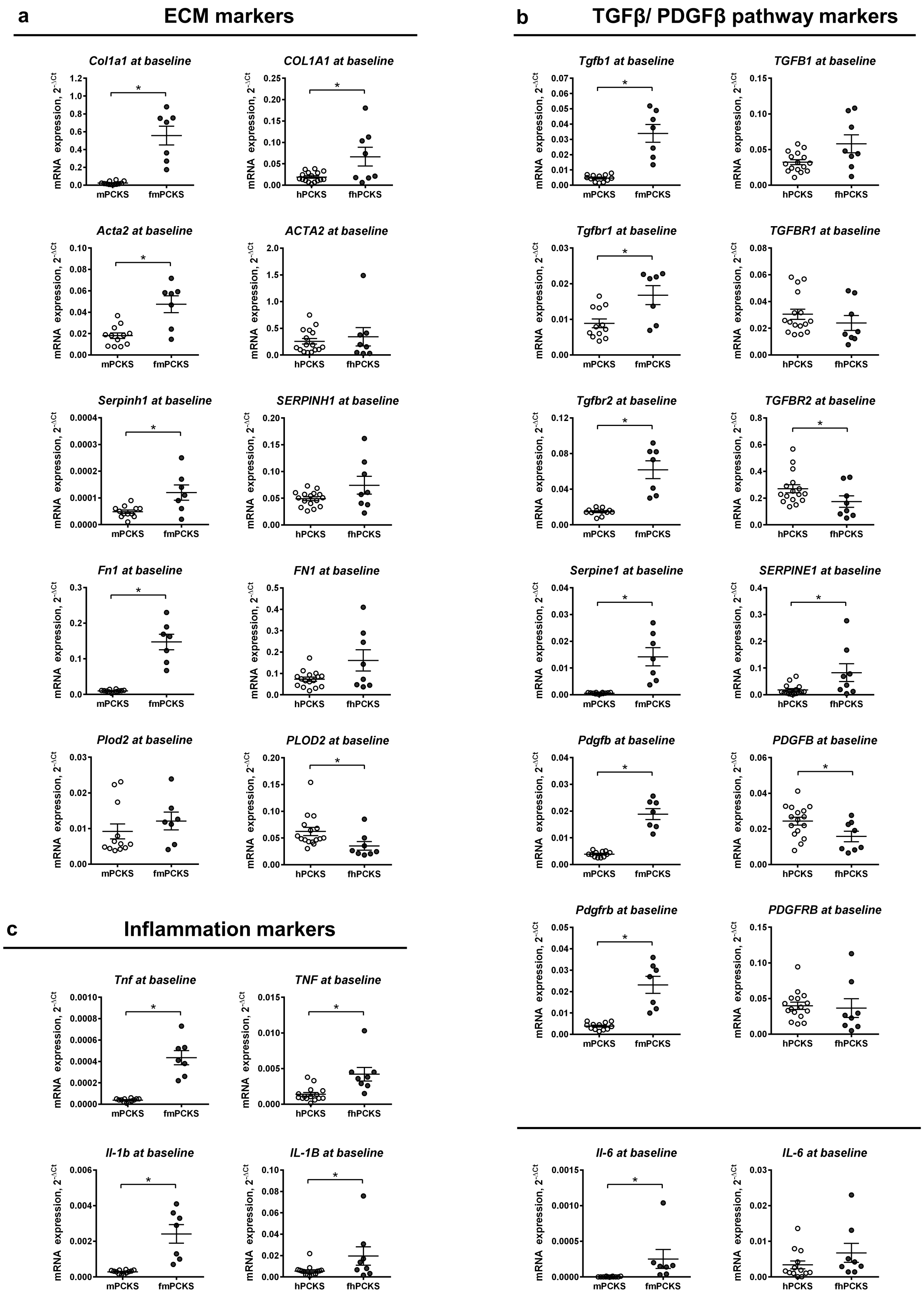

Figure S3. Baseline transcriptional profiles of murine and human PCKS showcasing the pre-existing diseased state of PCKS prepared from fibrotic kidneys prior culturing. PCKS were prepared from healthy murine and human kidneys (mPCKS and hPCKS) and from fibrotic kidneys (fmPCKS and fhPCKS). Graphs illustrate baseline (at 0h) mRNA levels of ECM markers (*COL1A1, ACTA2, SERPINH1, FN1* and *PLOD2*); markers of TGFβ and PDGF signaling (*TGFB, TGFBR1, TGFBR2, SERPINE1, PDGFB* and *PDGFRB*); and inflammation markers (*TNF, IL-1B* and *IL-6*). Data are expressed as mean ± SEM, n=7-12 (murine PCKS) or n=8-16 (human PCKS); *$p < 0.05$.

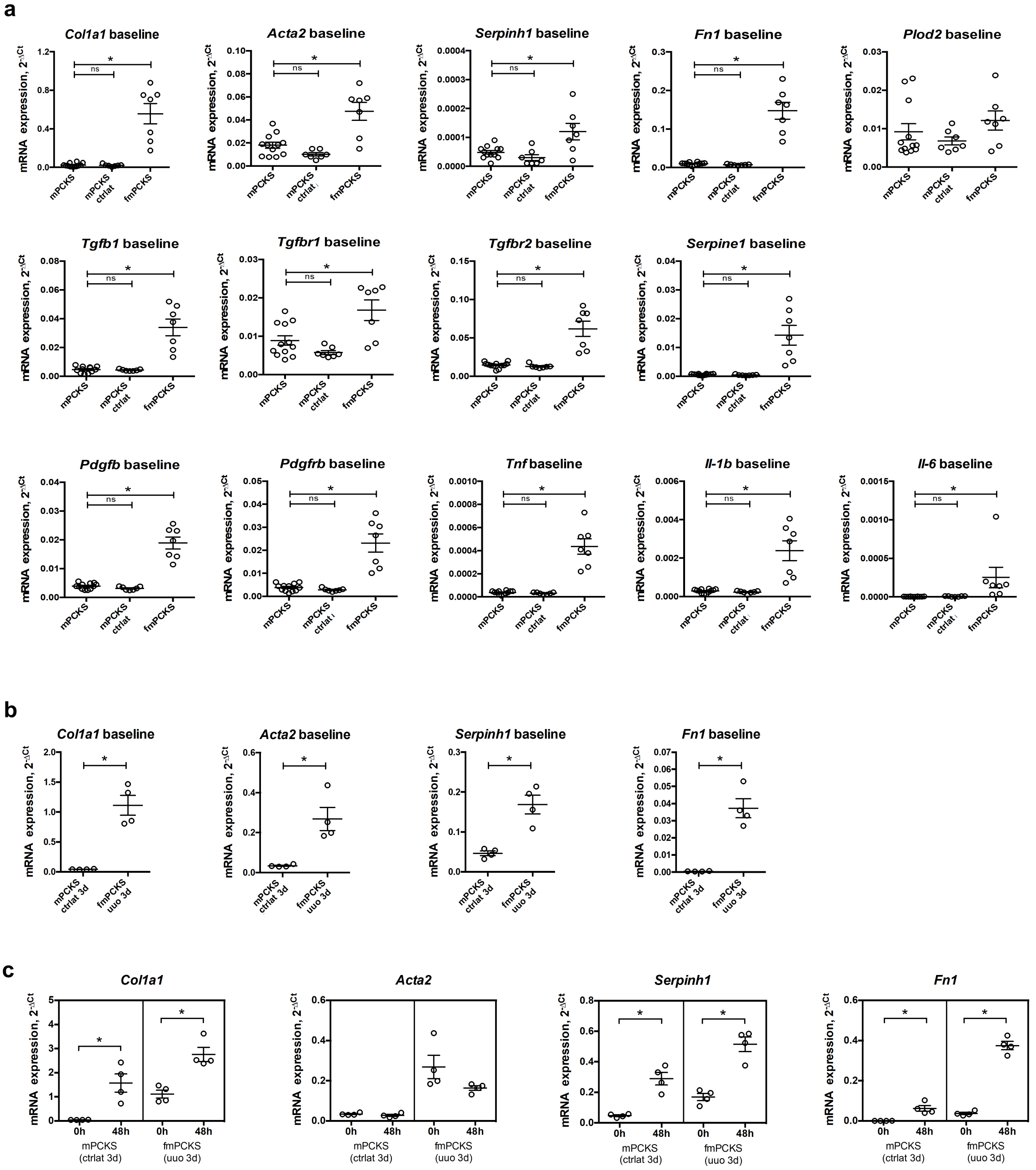

Figure S4. Baseline and culture-driven transcriptional regulation of fibrosis and inflammation markers in murine PCKS from healthy and UUO mice. Murine PCKS were prepared from healthy kidneys (mPCKS), from non-ligated, contralateral 7dUUO kidneys (mPCKS-ctrlat) and from fibrotic 7dUUO kidneys (fmPCKS). (a). Baseline mRNA levels of ECM markers (*Col1a1, Acta2, Serpinh1, Fn1* and *Plod2*); markers of TGFβ and PDGF signaling (*Tgfb1, Tgfbr1, Tgfbr2, Serpine1*, *Pdgfb* and *Pdgfrb*); and inflammation markers (*Tnf, Il-1b* and *Il-6*). Fibrotic slices (fmPCKS) showed clear diseased genotype, as they displayed high baseline levels for all tested transcripts compared to PCKS from healthy or non-ligated kidneys. In turn, mPCKS and mPCKS-ctrlat showed no differences in baseline expression of tested fibrosis and inflammation markers; therefore, mPCKS-ctrlat experimental group was omitted in this study. (b) Murine PCKS prepared from 3dUUO also displayed fibrotic genotype by highly expressing fibrosis markers already at 0h compared to PCKS from contralateral right kidneys. (c) Culture further induced mRNA expression of fibrosis markers in both 3dUUO mPCKS-ctrlat and fmPCKS. Data are expressed as mean ± SEM, n=3-5 (murine PCKS) or n=5-7 (human PCKS); *$p < 0.05$. UUO, unilateral ureteral obstruction.

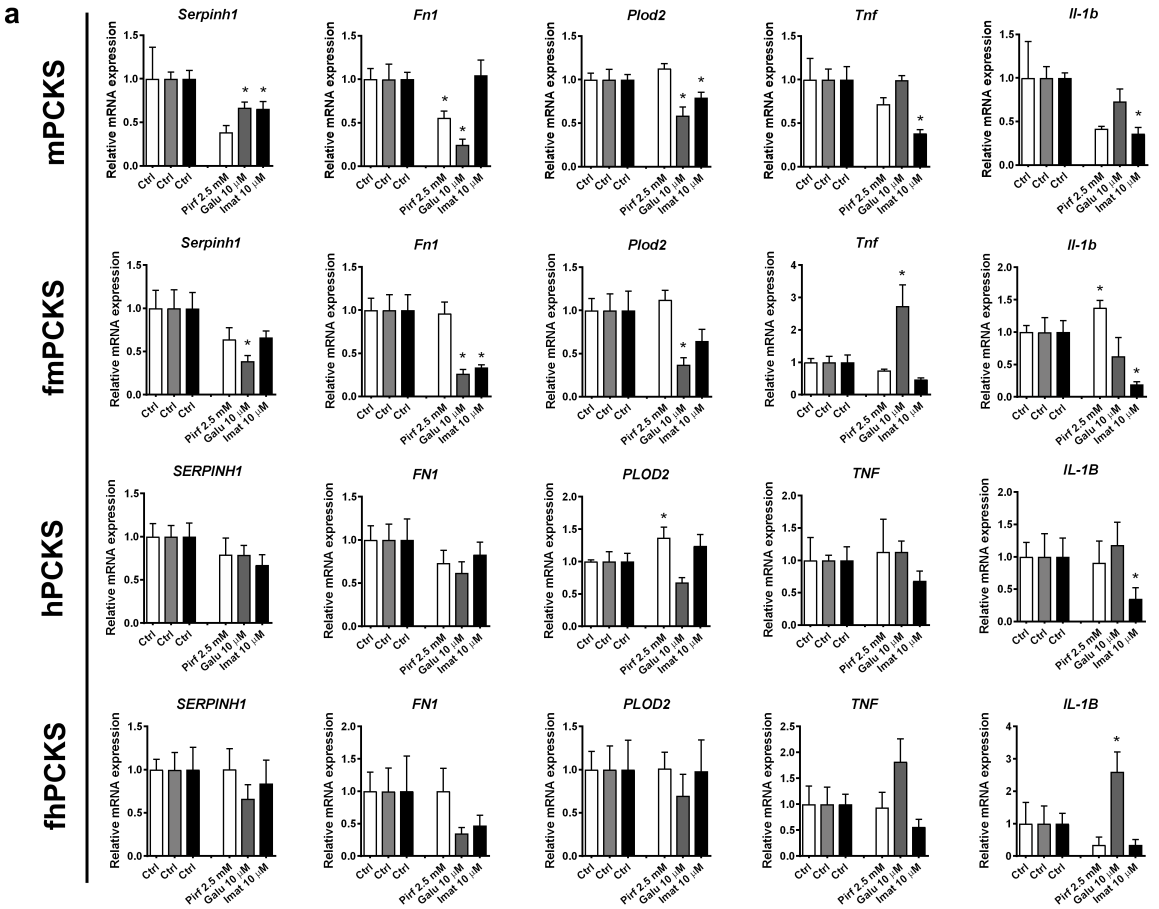
a
mPCKS
fmPCKS
hPCKS
fhPCKS
Serpinh1
Fn1
Plod2
Tnf
Il-1b
SERPINH1
FN1
PLOD2
TNF
IL-1B
Relative mRNA expression
Ctrl
Pirf 2.5 mM
Galu 10 μM
Imat 10 μM

b

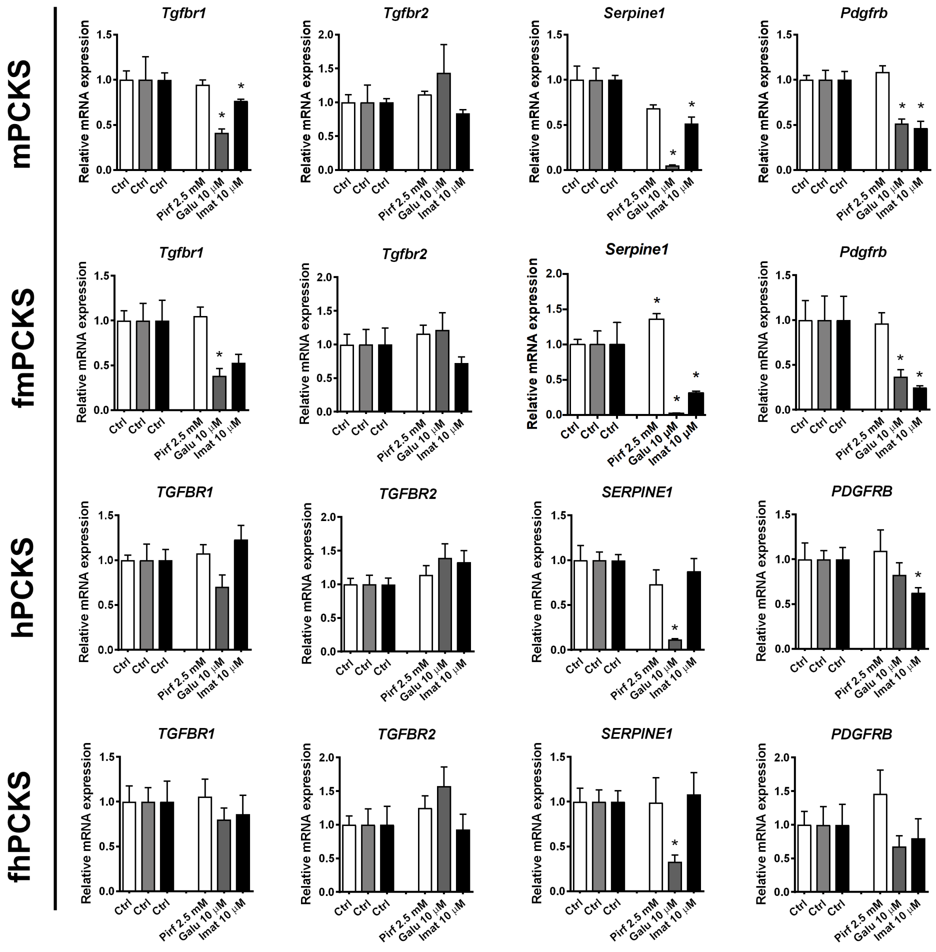

Figure S5. Complementary information on potency of tested anti-fibrotic compounds to attenuate culture-induced expression of fibrosis and inflammation markers in PCKS. Complementary information for Figure 3, as it illustrates the regulation of the rest of tested markers: ECM markers and inflammation markers (a), and markers of TGFβ and PDGF pathways (b). Data are expressed as mean ± SEM, n=3-5 (murine PCKS) or n=5-7 (human PCKS); *$p < 0.05$.

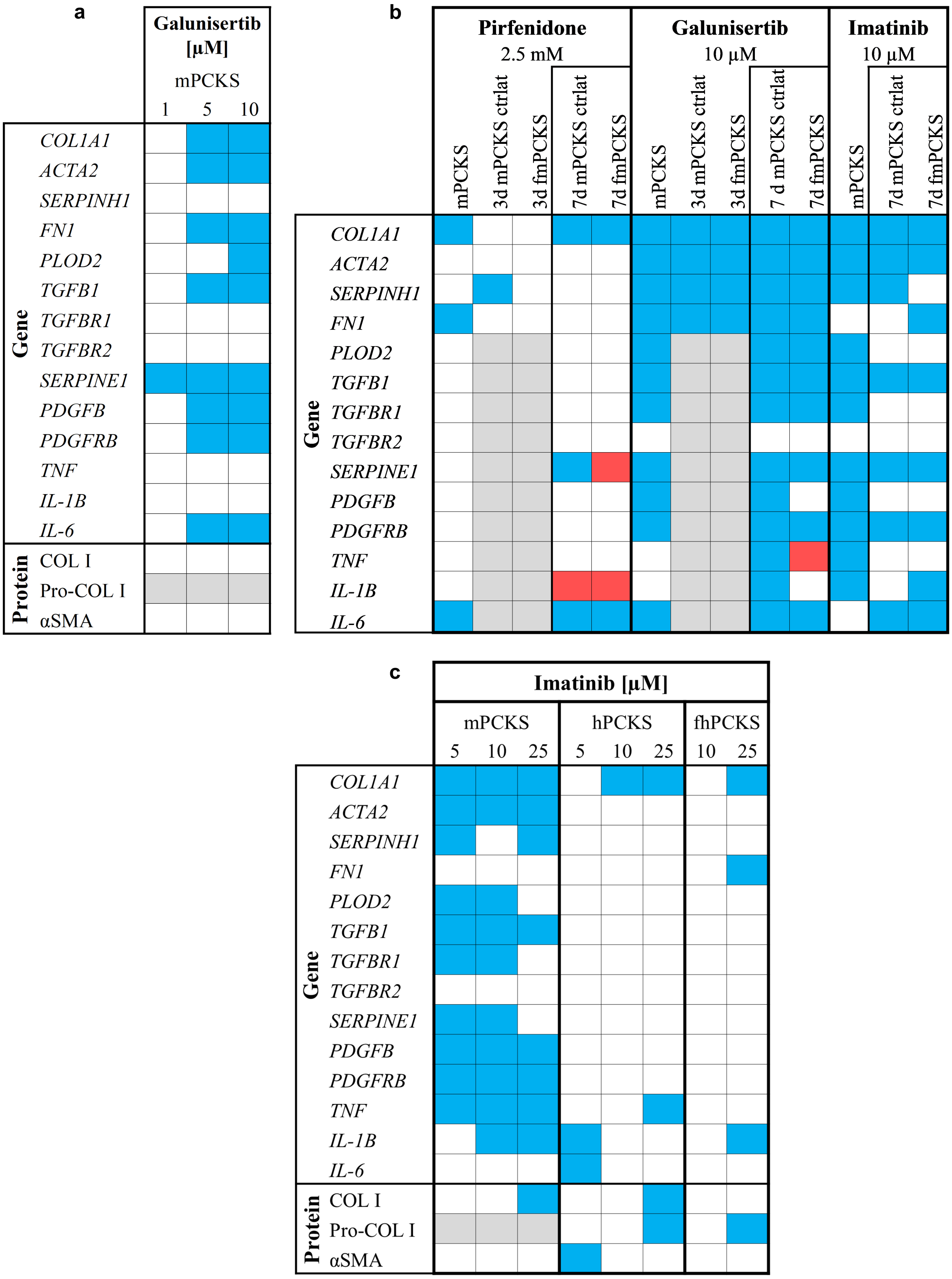

Figure S6. Visual summary of concentration-dependent effects of pirfenidone, galunisertib and imatinib in PCKS. (a) Changes in gene and protein expression of tested markers in murine healthy PCKS were treated with 1-10 µM galunisertib for 48h. (b) Transcriptional changes in murine healthy PCKS, PCKS prepared from non-ligated, contralateral kidneys (mPCKS-ctrlat) or fibrotic kidneys (fmPCKS) at 3d and 7d UUO, treated with pirfenidone 2.5 mM, galunisertib 10 µM or imatinib 10 µM for 48h. (c). Changes in gene and protein expression of tested markers in murine and human PCKS were treated with 5-25 µM imatinib for 48h. Blue color indicates a significant inhibition, red color shows significant increase, and markers that were not tested in any particular condition are colored in grey.

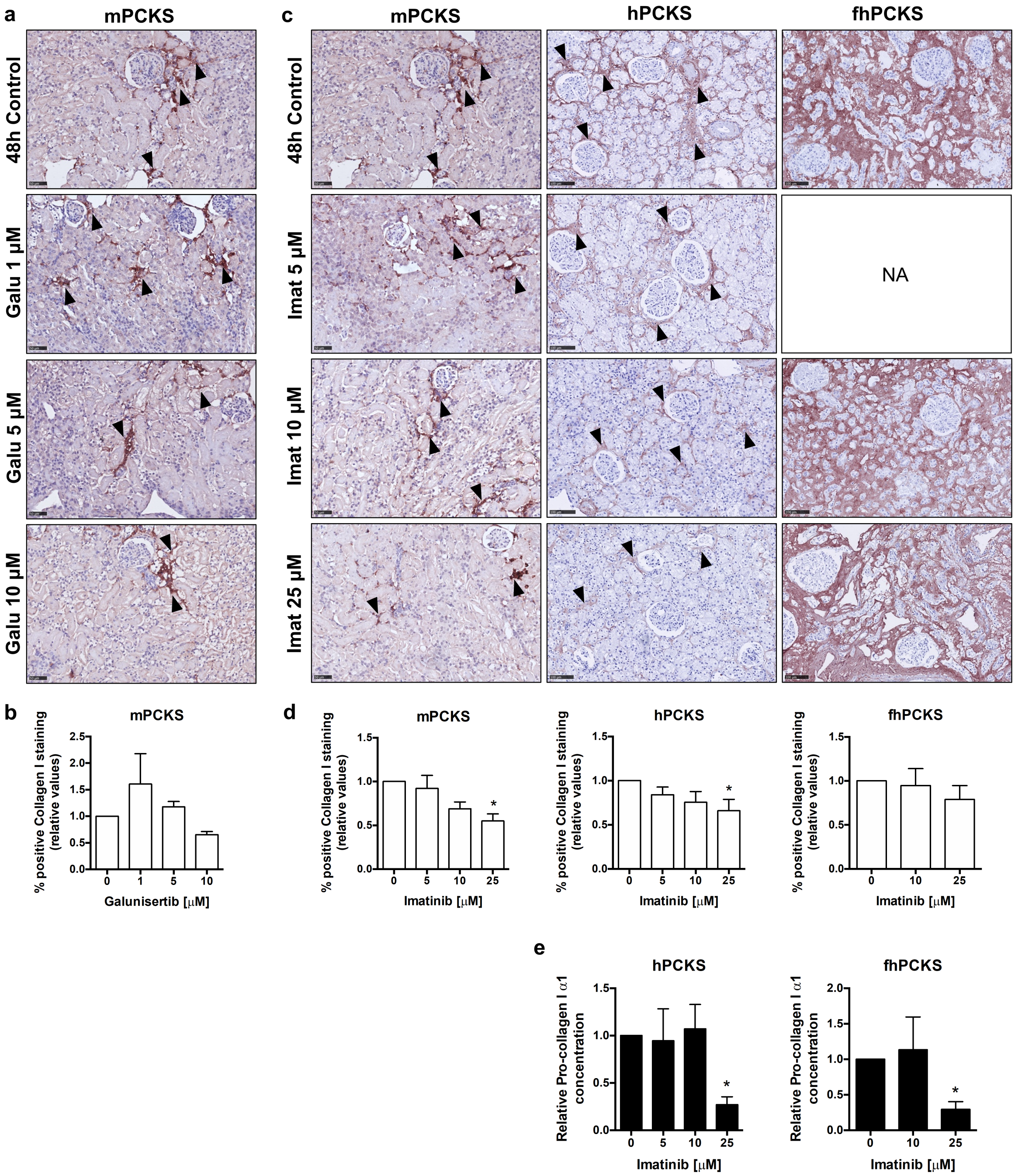

Figure S7. Concentration-dependent effects of galunisertib and imatinib on collagen type I deposition and its *de novo* biosynthesis in PCKS. Murine healthy control PCKS were treated with 1-10 µM galunisertib for 48h (a-b); murine and human PCKS were treated with 5-25 µM imatinib (c-d). Representative photomicrographs of immunohistochemistry for collagen type I in murine and human PCKS (a, c). Black arrow heads mark the collagen type I accumulation sites (also visualized by brown-red colour). Scale bars are 50 µm (murine PCKS) or 100 µm (human PCKS), and respective computerized quantitative analyses (b, d). Protein levels of procollagen type I (α1), a marker of newly synthesized collagen, secreted by human healthy and fibrotic PCKS treated with 5-25 µM imatinib for 48h, as measured by ELISA in culture supernatants (e). Data are expressed as mean ± SEM, n=3-5 (murine PCKS) or n=5-7 (human PCKS); *$p < 0.05$.

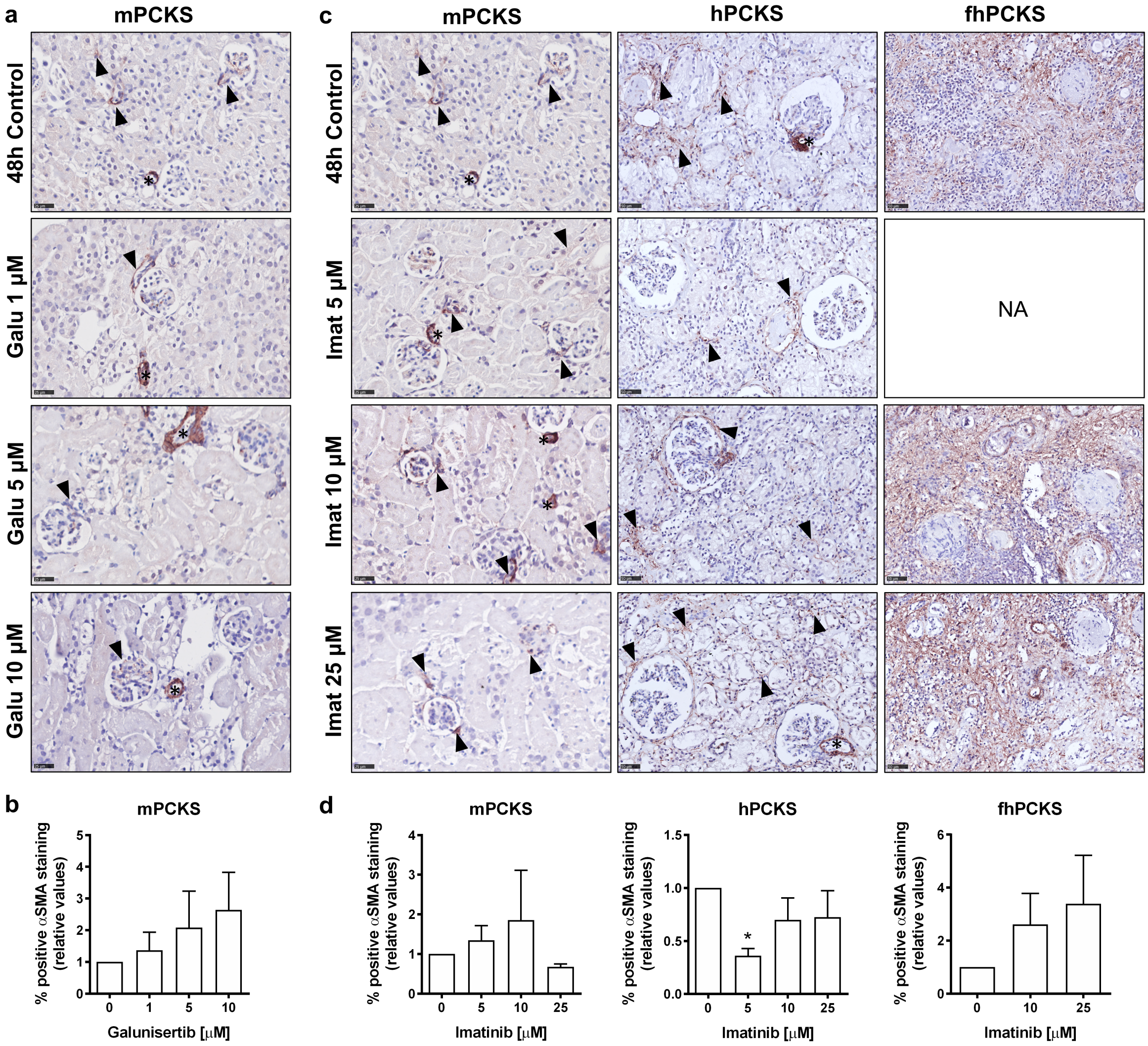

Figure S8. Concentration-dependent effects of galunisertib and imatinib on accumulation of alpha-smooth muscle actin (α-SMA) in PCKS. Murine healthy control PCKS were treated with 1-10 µM galunisertib for 48h (a-b); murine and human PCKS were treated with 5-25 µM imatinib (c-d). Representative photomicrographs of immunohistochemistry for α-SMA in murine and human PCKS (a, c). Black arrow heads mark the α-SMA expression sites (also visualized by brown-red colour). Stars indicate α-SMA-positive blood vessels that were excluded from the quantitative analysis. Scale bars are 25 µm (murine PCKS) or 50 µm (human PCKS), and respective computerized quantitative analyses (b, d). Data are expressed as mean ± SEM, n=3-5 (murine PCKS) or n=5-7 (human PCKS); *$p < 0.05$.

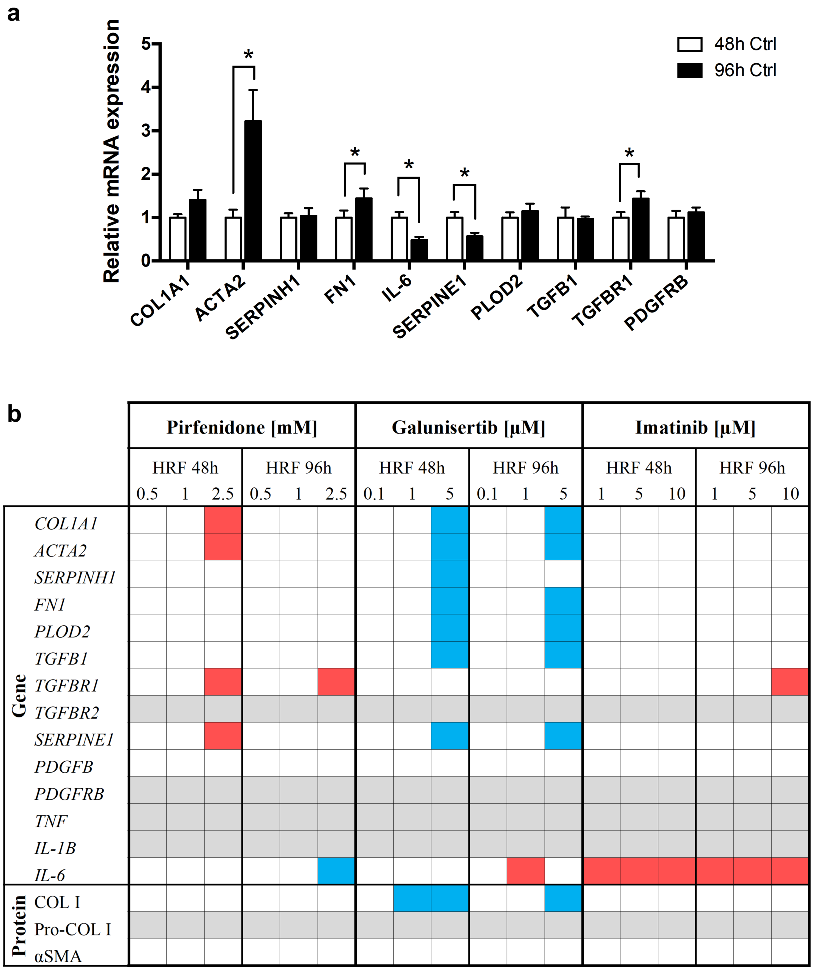

Figure S9. Transcriptional changes in human renal fibroblasts (HRFs) during culture and concentration-dependent anti-fibrotic effects of tested compounds. HRFs were stimulated with macromolecular crowder PVP-40 and TGFβ, and cultured for 48h and 96h. (a) mRNA expression levels of fibrosis and inflammation markers in HRFs at 96h compared to 48h. Data are expressed as mean ± SEM, n=3; *$p < 0.05$. (b) Summary of the observed anti-fibrotic effects of tested compounds in HRFs: fibroblasts were cultured in the presence of increasing concentrations of pirfenidone (0.5, 1, 2.5 mM), galunisertib (0.1, 1, 5 μM) or imatinib (1, 5, 10 μM) for 48h and 96h. Blue colour indicates a significant inhibition, red colour shows significant increase, and markers that were not tested in any particular condition are coloured in grey.
